# Activation of the PI3K/AKT signaling pathway by ARNTL2 enhances cellular glycolysis and sensitizes pancreatic adenocarcinoma to erlotinib

**DOI:** 10.1186/s12943-024-01965-5

**Published:** 2024-03-08

**Authors:** Weiyu Ge, Yanling Wang, Ming Quan, Tiebo Mao, Evelyne Y. Bischof, Haiyan Xu, Xiaofei Zhang, Shumin Li, Ming Yue, Jingyu Ma, Haiyan Yang, Lei Wang, Zhengyuan Yu, Liwei Wang, Jiujie Cui

**Affiliations:** 1 Department of Oncology and State Key Laboratory of Systems Medicine for Cancer of Shanghai Cancer Institute, Renji Hospital, School of Medicine, Shanghai Jiaotong University, Shanghai, 200127 China; 2 Department of Medical Oncology, Shanghai Medical College, Fudan University Shanghai Cancer Center, Fudan University, Shanghai, 200032 People’s Republic of China; 3 Department of Oncology and Tumor Institute, Shanghai East Hospital, Tongji University School of Medicine, Shanghai, China; 4 Department of Oncology, The Affiliated Lianyungang Hospital of Xuzhou Medical University, The First People’s Hospital of Lianyungang, Jiangsu, China; 5 Department of Medical Oncology, The First Affiliated Hospital of Soochow University, Suzhou, Jiangsu China

**Keywords:** Pancreatic adenocarcinoma, ARNTL2, Prognosis, Erlotinib, Targeted therapy, precision oncology

## Abstract

**Background:**

Pancreatic adenocarcinoma (PC) is an aggressive malignancy with limited treatment options. The poor prognosis primarily stems from late-stage diagnosis and when the disease has become therapeutically challenging. There is an urgent need to identify specific biomarkers for cancer subtyping and early detection to enhance both morbidity and mortality outcomes. The addition of the EGFR tyrosine kinase inhibitor (TKI), erlotinib, to gemcitabine chemotherapy for the first-line treatment of patients with advanced pancreatic cancer slightly improved outcomes. However, restricted clinical benefits may be linked to the absence of well-characterized criteria for stratification and dependable biomarkers for the prediction of treatment effectiveness.

**Methods and results:**

We examined the levels of various cancer hallmarks and identified glycolysis as the primary risk factor for overall survival in PC. Subsequently, we developed a glycolysis-related score (GRS) model to accurately distinguish PC patients with high GRS. Through in silico screening of 4398 compounds, we discovered that erlotinib had the strongest therapeutic benefits for high-GRS PC patients. Furthermore, we identified ARNTL2 as a novel prognostic biomarker and a predictive factor for erlotinib treatment responsiveness in patients with PC. Inhibition of ARNTL2 expression reduced the therapeutic efficacy, whereas increased expression of ARNTL2 improved PC cell sensitivity to erlotinib. Validation in vivo using patient-derived xenografts (PDX-PC) with varying ARNTL2 expression levels demonstrated that erlotinib monotherapy effectively halted tumor progression in PDX-PC models with high ARNTL2 expression. In contrast, PDX-PC models lacking ARNTL2 did not respond favorably to erlotinib treatment. Mechanistically, we demonstrated that the ARNTL2/E2F1 axis-mediated cellular glycolysis sensitizes PC cells to erlotinib treatment by activating the PI3K/AKT signaling pathway.

**Conclusions:**

Our investigations have identified ARNTL2 as a novel prognostic biomarker and predictive indicator of sensitivity. These results will help to identify erlotinib-responsive cases of PC and improve treatment outcomes. These findings contribute to the advancement of precision oncology, enabling more accurate and targeted therapeutic interventions.

**Supplementary Information:**

The online version contains supplementary material available at 10.1186/s12943-024-01965-5.

## Background

Pancreatic adenocarcinoma (PC) is a highly malignant tumor. Its incidence has been rapidly increasing, but there has been limited progress in its early diagnosis and the development of an effective therapy over the past decade [1, 2]. Since there are currently no validated efficient targeted therapies for PC, chemotherapy remains the main treatment approach [3, 4]. The management of PC would greatly benefit from reliable predictive and prognostic biomarkers, as well as strategic cancer stratification and standardization [5]. Several large randomized studies have indicated that most patients who undergo surgery and receive adjuvant therapy do not experience significant overall survival benefits, particularly among unselected patients [6]. Thus, there is an urgent need to identify biomarkers and new effective targets that can better select patients for established combination therapies. The human epidermal growth factor receptor type 1 (HER1/EGFR) is overexpressed in pancreatic cancer and associated with poor prognosis and tumor progression [7, 8]. Consequently, blocking HER1/EGFR tyrosine kinase signaling has been found to inhibit the growth and metastasis of human pancreatic tumor xenografts [9] and enhance the antitumor effects of gemcitabine [10].

Erlotinib is an oral inhibitor of the HER1/EGFR tyrosine kinase, currently approved for patients with non-small cell lung cancer [11]. FDA approval for pancreatic cancer (PC) patients was granted based on the statistically significant improved overall survival rate when gemcitabine was combined with erlotinib, compared to gemcitabine monotherapy [12, 13]. However, the use of erlotinib for advanced pancreatic cancer is restricted due to limited clinical response. Therefore, it is essential to identify and characterize novel targets for patient stratification. Furthermore, the discovery of biomarkers as predictors to identify pancreatic cancer patients who would specifically benefit from erlotinib therapy is important.

ARNTL2, which encodes a basic helix-loop-helix transcription factor called aryl hydrocarbon receptor nuclear translocator like 2, is a biologically relevant partner of circadian and hypoxia factors [14, 15]. Growing evidence suggests that ARNTL2 promotes tumor cell migration, invasion, and metastasis in various cancers, including breast, colon, lung, and pancreatic cancers [16–20]. However, the clinical implications and association of ARNTL2 with the effectiveness of targeted therapy in pancreatic cancer have not been reported.

In this study, we identified a pivotal classification criterion with prognostic potential for patients with pancreatic cancer and discovered an effective biomarker for the triage of PC patients who could benefit from erlotinib-targeted therapy. We demonstrate that glycolysis, a metabolic pathway, is associated with decreased overall survival in PC. We established a gene signature related to glycolysis for predicting PC prognosis. Importantly, we found that a high glycolysis-related gene signature (GRS) and higher expression level of ARNTL2 were significantly correlated with a better response to erlotinib treatment in pancreatic cancer patients. This correlation was confirmed through a series of in vitro assays and in vivo validation using patient-derived xenograft (PDX) models of pancreatic cancer. Mechanistically, we demonstrate that ARNTL2-mediated glycolysis enhanced erlotinib responsiveness through activation of the PI3K/AKT pathway in pancreatic cancer. Altogether, our findings identify ARNTL2 as a novel prognostic biomarker and a sensitivity predictor enabling the identification of erlotinib-sensitive pancreatic cancer patients.

## Materials and methods

### Data source and data processing

RNA-seq expression profiles and clinical data of 160 TCGA-PAAD patients were downloaded and collected from The Cancer Genome Atlas (TCGA, https://xenabrowser.net/datapages/) as the training set. Transcriptome data of 101 PC patients were retrieved from the International Cancer Genome Consortium portal (ICGC, https://cc.icgc.org/projects/LIRI-JP) to serve as the testing set. In addition, RNA expression data from 167 healthy pancreatic tissues were obtained from the public database Genotype-Tissue Expression Portal (GTEx, https://gtexportal.org/home/). We excluded samples from patients lacking important clinicopathological or survival information.

### Glycolysis-related risk score (GRS), biomarker selection and signatures

To identify differences in biological functions in PC patients, the performances of cancer hallmarks in the TCGA cohort were estimated by a single-sample gene set enrichment analysis (ssGSEA) algorithm (R package ‘gsva’) based on RNA-seq expression profiles and hallmark gene signatures from the Molecular Signatures Database (MSigDB) [21]. The significance of different cancer hallmarks in PC was assessed by a univariate Cox proportional-hazards (Cox-PH) regression model. Weighted gene co-expression network analysis (WGCNA) was used to establish a scale-free co-expression network using the R package ‘WGCNA’ [20] and to determine a gene module that is mostly related to glycolysis [22]. Additionally, we further acquired the most robust prognostic markers by a least absolute shrinkage and selection operator (LASSO) Cox regression model [23].

### Cell culture

The PC cell lines (CFPAC-1, PANC-1, AsPC-1, BxPC3, and PATU-8988 T), and normal pancreatic duct cell lines (HPNE) were obtained from the Chinese Academy of Science (Shanghai, China). CFPAC-1 is maintained in IMEM medium (Gibco, NY, USA), and HPNE and PANC-1 are grown in DMEM medium supplemented with 10% FBS (Gibco, NY, USA) and 100 U/ml penicillin/streptomycin (Corning, NY, USA) in a humidified incubator under a 5% CO_2_ atmosphere at 37 °C. AsPC-1, PATU-8988 T grown in RPMI 1640 medium (HyClone) supplemented with 1% penicillin–streptomycin, 10% FBS.

### Plasmids and lentivirus production

For the lentivirus packaging, ARNTL2 and E2F1 shRNAs and ARNTL2 overexpression vectors were obtained from Qin ke Company (Shanghai, China). Lentivirus encoding ARNTL2- knockdown and E2F1-knockeown shRNA, and ARNTL2-overexpression plasmids, and an empty lentivirus were transformed into PC cells (5 × 10^5^) for 48 h, and then selected by puromycin treatment (Santa Cruz Biotechnology, CA, USA) for 1–2 week.

### Western blotting

Total proteins were prepared using RIPA lysis buffer (Beyotime, China). Proteins were quantified by BCA assay. An equal amount of proteins was loaded to gel and separated by SDS-PAGE using the NuPAGE Novex Midi Gel system on 4% to 12% Bis–Tris gels (Share-bio, SB-FP11420), then transferred to a polyvinylidene difluoride membrane, blocked and tested with primary antibodies at 4 °C overnight, and with horseradish peroxidase-conjugated secondary antibody (Santa Cruz Biotechnology).

### RNA extraction and quantitative RT-PCR (qRT-PCR)

Total RNA from the PC cell lines was extracted using a ZEB kit (Ebioscience). RNA was used for cDNA synthesis with the Superscript III Reverse Transcription Reagent (Life Technologies). The real-time PCR reaction was performed according to the protocol of the SYBR Premix Ex Taq kit (Takara) and using a StepOnePlus Real-Time PCR System (Applied Biosystems, USA). Primers are shown in Table S1.

### Glycolytic rate assay

The PC cells were seeded at a density of 1 × 10^5^/well into an XF96 plate. The glycolysis capacity of the PC cells was investigated using the Seahorse XF Glycolysis Stress Test Kit according to the manufacturer. Glucose, Oligomycin, and 2-DG were diluted into Seahorse XF DMEM and loaded into the accompanying cartridge to achieve the desired final concentrations. Injections of the drugs occurred at the time points specified in the figure legends.

### Clone formation experiment

Cells were plated into 6-well plates at a density of 5 × 10^4^ cells per well and cultured in the medium containing the indicated drugs for 7–14 days (the medium was changed three times a week). Cells were fixed with 4% formaldehyde in PBS and stained with 0.1% crystal violet diluted in water. Cell confluency in each well was quantified using Image J software.

### Cell proliferation assays

The PC cell lines (CFPAC-1, PANC-1, AsPC-1, PATU-8988 T), and normal pancreatic duct cell line (HPNE) were treated with erlotinib alone for 72 h. To analyze the cell growth rate, 4 × 10^3^ cells in 200 ul were grown in a 96-well plate for 72 h. Cell proliferation was evaluated every 24 h using the Cell Counting Kit-8 (CCK-8) Test (Beyotime, China).

### Flow cytometry

PANC-1 and PATU-8988 T cells were seeded in 6-well plates overnight. After the indicated treatments, cells were collected using trypsin, washed twice with phosphate-buffered saline, and stained using the Annexin-V-APC-633/PI Apoptosis Detection Kit (AD10/11, DOJINDO, Japan) according to the manufacturer’s instructions. Stained cells were analyzed using a FACSCelesta™ multicolor flow cytometer (Becton, Dickson and Company, USA), and data were processed using FlowJo software (Becton, Dickson and Company, USA).

### ﻿Chromatin immunoprecipitation (ChIP) assay

2 × 10^6^ PANC-1 cells were prepared for the ChIP assay. The ChIP assay kit was got from EMD Millipore (Billerica, MA, USA). The resulting precipitated DNA specimens were tested using qRT-PCR to amplify a 171-bp region of the ARNTL2 promoter and the primers are shown in Table S1.

### PC-PDX tumor models

Fresh pancreatic ductal adenocarcinoma tissue was obtained from patients who underwent surgical resection at Shanghai Renji Hospital with approval by the Ethics Committee of Renji Hospital, affiliated with Shanghai Jiao Tong University School of Medicine (Shanghai, China, KY[2019]035). Two types of pancreatic cancer tissues were selected for immunohistochemistry: two samples with high expression of ARNTL2 and two with low expression of ARNTL2. Once samples were obtained, they were trimmed, cut into 10–20 mm^3^ fragments, and subcutaneously transplanted into the flanks of 6-week-old female BALB/c nude mice from Shanghai Laboratory Animal Center and allowed to grow as the first passage. Once the tumor burden reached about 1000 mm^3^, mice were euthanized, tumors collected and cut into 50 mg pieces, and then serially propagated in additional mice. The excess portion of the tissue from the first passage was snap-frozen. Implantation of subsequent passages provided stable and comparable xenografts, and fragments from third generation (P3) passage of PDXs were then surgically implanted for subsequent experiments. Specific informed consent for PDX model generation was obtained from all patients (V1.0, Dec. 26th, 2018). Additionally, all animal experiments were approved by the Animal Ethics Committee of Shanghai Renji Hospital, Shanghai Jiao Tong University School of Medicine (Shanghai, China, RTMS-20220601(03)).

### Efficacy evaluation of erlotinib treatment in PDX tumor models

Once the tumor volume of PC-PDX(P3) reached 60–100 mm^3^, mice were randomly assigned to the vehicle and erlotinib treatment groups (Selleck Chemicals, 100 mg/kg in H_2_O containing 5% DMSO, 40% PEG 400, and 5% Tween-80 by gastric gavage once daily for two weeks). Tumors were measured and monitored every two days. Tumor volume based on caliper measurements was estimated using the modified ellipsoidal formula: tumor volume = ½ length × width^2^. All control animals were dosed with an equal volume of the vehicle. Finally, the tumor tissues were collected, weighed, and photographed at the end of the experiments.

### Estimation of drug response in clinical samples

Expression profile data of human cancer cell lines (CCLs) were obtained from the Broad Institute Cancer Cell Line Encyclopedia (CCLE) project (https://portals.broadinstitute.org/ccle/) [24]. Drug sensitivity data of human cancer cell lines (CCLs, https://portals.broadinstitute.org/ccle/) were sourced from the Cancer Target Discovery and Development (CTD^2^) Network (CTD^2^, https://ocg.cancer.gov/programs/ctd2/data-portal) [25]., PRISM Repurposing dataset (PRISM, https://depmap.org/portal/prism/) and Genomics of Drug Sensitivity in Cancer (GDSC, https://depmap.org/portal/prism/). The CTD^2^ contains sensitivity data for 401 compounds over 816 CCLs, the PRISM for 4481 compounds over 569 CCLs, and GDSC for 222 compounds over 987 CCLs. These datasets provide the area under the dose–response curve (area under the curve-AUC) values as a measure of drug sensitivity, and lower AUC values indicate increased sensitivity to treatment. K-nearest neighbor (k-NN) imputation was applied to impute the missing AUC values. Before imputation, compounds with more than 20% of missing data were excluded. Because the CCLs in both datasets were obtained from the CCLE project, molecular data in CCLE were thus used for subsequent CTD^2^, PRISM, and GDSC analyses [26]. Estimation of drug response in clinical samples was performed using the pRRophetic R package.

### Additional statistical analyses

Time-dependent receiver operating characteristic (tROC) analysis was performed to measure the predictive power with the R package ‘survival ROC’ [27]. The Kaplan–Meier method was used to draw survival curves, and the log-rank test was applied to estimate differences. Student’s t-test or one-way analysis of variance (ANOVA) was performed to analyze differences between groups in variables. A *p*-value < 0.05 was considered statistically significant. In addition, IBM SPSS Statistics 20, GraphPad Prism 9.0, and 3.6.4.R software were applied to analyze data and plot graphs.

## Results

### Glycolysis represents a primary risk factor for overall survival in PC patients

**Fig. 1:**
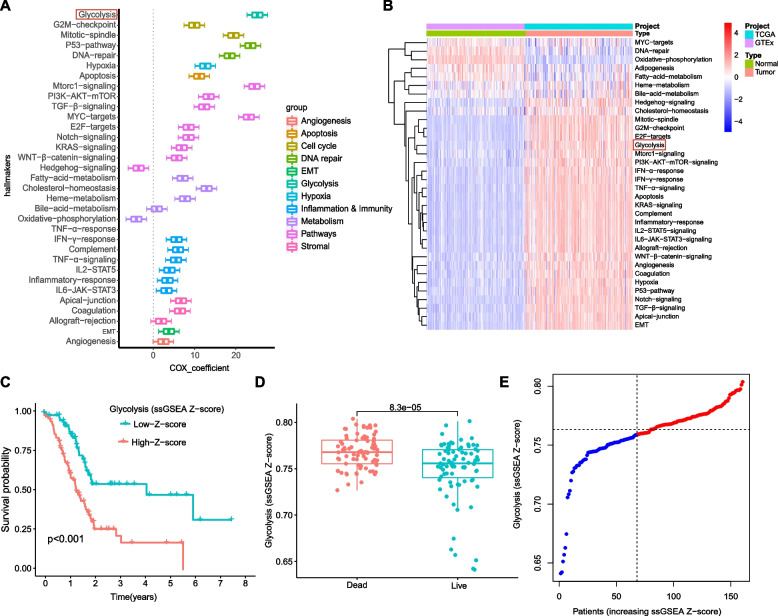
Glycolysis is identified as the primary risk factor for survival. **A** Univariate Cox regression analysis indicated that glycolysis was the primary risk factor among various hallmarks of cancer; **B** Heatmap showing differentially enriched biological pathways between normal and PC tumor tissues; **C** Kaplan–Meier analysis showed that patients with higher ssGSEA scores of glycolysis displayed worse OS; **D**-**E** Glycolysis ssGSEA scores were significantly elevated in patients who died during follow up. Survival difference was compared using the log-rank test

We estimated and ranked the Cox coefficients for each cancer hallmark, and among them, glycolysis was aberrantly hyperactivated in tumor tissues (Fig. 1A) and exhibited the strongest impact on the survival of pancreatic cancer (PC) patients (Fig. 1B). We next divided 160 PC patients into two groups based on the median Z-score and found that patients with lower median Z-scores had significantly longer OS (Fig. 1C). Furthermore, our data demonstrated markedly higher Z-scores of the glycolysis ssGSEA in patients who were deceased at the follow-up compared with living patients at the same time point (Fig. 1D-E). These findings indicate that glycolysis is a strong prognostic factor associated with reduced overall survival in PC patients.

### Construction of a glycolysis-related gene signature

To identify a module most correlated with glycolysis, we performed the WGCNA analysis of a total of 14 non-grey generated modules (Figure S1A, Table S2). Magenta module showed the highest relativity (*r* = 0.37, *p* = 8e − 06) and correlation with glycolysis (Figure S1B). Using a threshold of p-value for gene significance (GS) less than 0.0001 and a *p*-value for univariate Cox regression below 0.05, we identified 335 hub genes from the 'glycolysis module' (Table S3). We next selected the optimal prognostic signature with the LASSO regression analysis to further screen for the most robust prognostic markers and identified two hub biomarkers (TPX2 and ARNTL2). A glycolysis prognostic model was constructed based on the expression of TPX2 and ARNTL2. Due to the risk score derived from the glycolysis-related gene signature, it was defined as the glycolysis-related score (GRS): GRS = ∑(0.997* expression of ARNTL2) + (0.727* expression of TPX2). In the TCGA cohort, the survival rate was higher among those with a low GRS when compared to those with a high GRS (Figure S1C). As a result of the correlations between GRS and patients’ prognosis, we incorporated clinical parameters to plot a nomogram and estimate 1-, 2-, and 3-year OS for PC patients (Figure S1D). The distribution of LASSO coefficients of the gene signature is shown in Figure S1E and the upregulated expression of ARNTL2 and TPX2 in the GRS high group is shown in Figure S1F.

Furthermore, we assessed the AUC values of these clinical parameters as predictors of the OS. As shown in Figure S1G, the AUC values confirmed an excellent predictive ability of the nomogram. Altogether our results demonstrate that GRS is a reliable predictive factor in PC patients.

### Assessment of the prognostic value of GRS within subgroups

For Kaplan–Meier survival analysis, patients in TCGA and ICGC cohorts were divided into high and low GRS groups according to the median value of GRS. In both cohorts, a consistent difference was observed between the two groups, patients with higher GRS exhibited worse prognosis than those with lower GRS (Figure S2A-B). Among various clinicopathological variables, univariate and multivariate Cox regression modeling indicated that only GRS was an independent risk factor for overall survival in the two cohorts (Figure S2C-D). To further investigate whether GRS has a superior predictive power for overall survival in PC patients, time-dependent ROC analysis was performed (Figure S2E-F). Results from these studies suggest that GRS maintains a good predictive performance in various PC cohorts.

### Identification of candidate agents with higher drug sensitivity in high-GRS PC patients

**Fig. 2:**
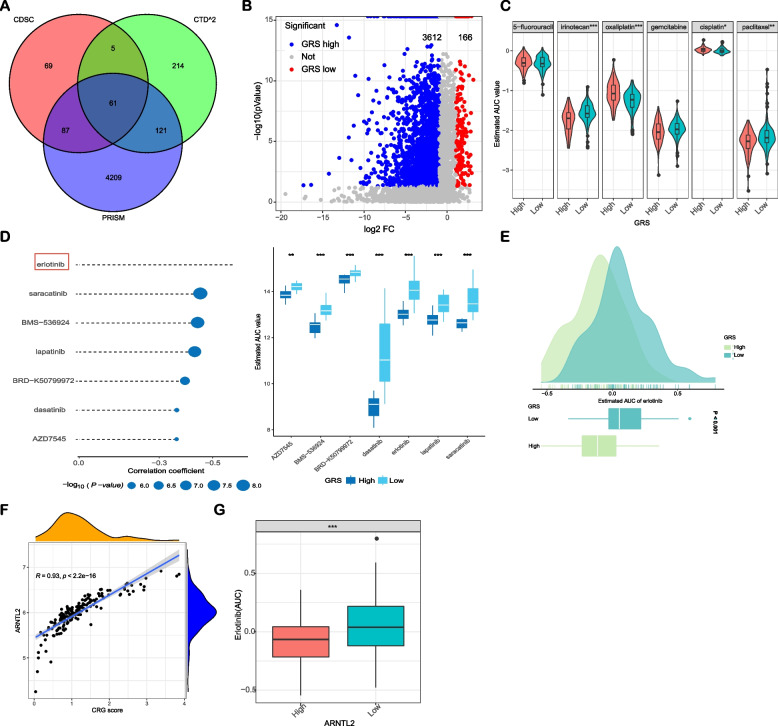
Identification of erlotinib with higher drug sensitivity in PC patients with high-GRS. **A** Venn diagram for summarizing included candidate agents from CTD^2^, GDSC, and PRISM datasets; **B** Volcano plot of differential candidate agents between low-GRS and high-GRS groups. (Wilcoxon rank-sum test: adjust *p* < 0.05, and log2FC > 1); **C** The differential drug response analysis of current treatment for advanced pancreatic cancer; **D** The results of Spearman’s correlation analysis and differential drug response analysis of seven candidate agents; **E** Comparison of estimated erlotinib’s sensitivity (logAUC) between low-GRS and high-GRS groups; **F** Relationship between erlotinib’s sensitivity and the expression of ARNTL2 in PC; **G** Expression level of ARNTL2 between low-GRS and high-GRS groups

The GDSC, CTD^2^, and PRISM datasets contain gene expression and drug sensitivity profiles of hundreds of CCLs, which can be utilized to construct a prediction model of drug response. After removing duplicated and NA, there were 4398 compounds in total (Table S4), with overlap in 61 compounds (Fig. 2A). To assess the utility of GRS as a predictive biomarker for therapy response in pancreatic cancer (PC) patients, we conducted analyses using drug response data obtained from the datasets. First, differential drug response analysis between GRS-high and GRS-low groups was performed to identify compounds with lower estimated AUC values in the high-GRS group (log_2_FC > 1, *p*-value < 0.05) (Fig. 2B). Importantly, we also selected chemotherapy drugs currently used for the treatment of advanced pancreatic cancer to estimate the sensitivities of patients in the low- and high-GRS groups to these drugs. We observed that patients with low GRS may positively react to oxaliplatin and cisplatin, while patients with high GRS may respond better to irinotecan and paclitaxel (Fig. 2C).

Consequently, Spearman correlation analysis between AUC value and GRS was used to select compounds with a negative correlation coefficient (Spearman’s r < -0.40). These analyses yielded seven candidate compounds (including erlotinib, saracatinib, BMS-536924, AZD7545, dasatinib, BRD-K50799972, and lapatinib), which shows a higher drug sensitivity in GRS-high patients (Fig. 2D), among them, erlotinib has maximum correlation coefficient.

### Erlotinib sensitivity in high-GRS PC patients

The analyses mentioned above, in isolation, did not provide sufficient evidence to conclude that these compounds exhibited therapeutic effects on PC. Considering that erlotinib is the only targeted drug approved by the FDA for advanced pancreatic cancer [12], we further assessed the AUC values of erlotinib between low- and high-GRS groups. Results indicated that patients with low GRS display significantly higher estimated AUC values (*p* < 0.001) suggesting a potential therapeutic benefit of erlotinib in PC patients with high GRS (Fig. 2E).

### The functional implications of ARNTL2 with EGFR signaling in PC

**Fig. 3:**
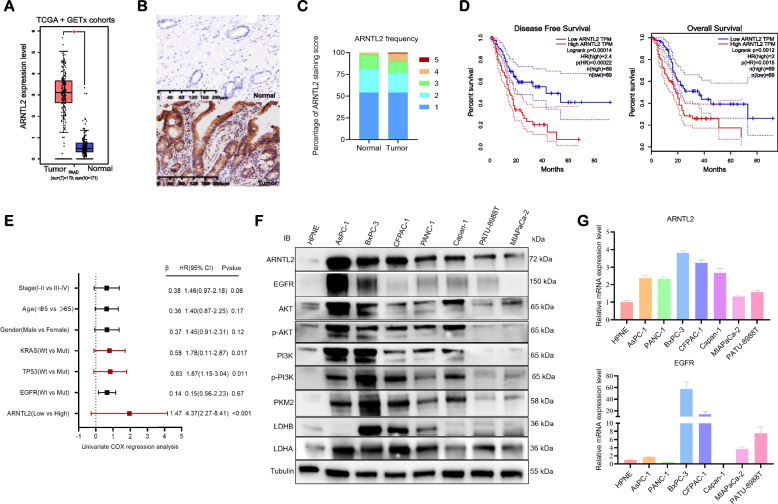
The functional implications of ARNTL2 and its relationship with EGFR in PC. **A** mRNA expression of ARNTL2 in PC from TCGA cohort and normal tissues from GTEx cohort; **B** Protein expression of ARNTL2 in PC and normal tissues; **C** IHC score of ARNTL2 in pancreatic cancer TMAs; **D** Kaplan–Meier survival analysis of the correlation between ARNTL2 expression and OS and DFS of PC patient in TCGA cohort; **E** The univariate Cox regression analysis were performed to depict the correlations between ARNTL2 expression and the clinicopathological features; **F** The protein expression levels of ARNTL2, EGFR and EGFR downstream signaling PI3K/AKT signaling pathway in PC cell lines (AsPC-1, BxPC-3, PANC-1, CFPAC-1, Capan-1, PATU-8988 T and MIAPaCa-2) and normal pancreatic duct cells (HPNE) were determined by western blot analysis; **G** The mRNA expression levels of ARNTL2 and EGFR in PC cell lines and normal pancreas cells were determined by qRT-PCR analysis. All data are presented as the mean ± SEM of triplicate experiments. *p < 0.05 by repeated measures with Student’s t-test

GRS which was established based on the expression of ARNTL2 and TPX2 showed the highest positive correlation with the expression of ARNTL2 (R = 0.93, *p* < 0.001) and ARNTL2 was highly expressed in PC patients with high GRS (Fig. 2F and S1D). Furthermore, a strong negative correlation between the expression level of ARNTL2 and the AUC value of erlotinib was identified (Fig. 2G). Next, we analyzed the ARNTL2 transcription expression in PC from TCGA and GTEx data sets. ARNTL2 was significantly upregulated in tumor tissues when compared to normal tissues (Fig. 3A). Consistent with these results, tissue microarrays (TMA) also revealed high expression of ARNTL2 protein levels in PC tissues when compared to normal pancreatic tissues (Fig. 3B-C). Moreover, we found that the high expression of ARNTL2 in PC was significantly correlated with worse overall survival and disease-free survival (Fig. 3D).

As a HER1/EGFR tyrosine kinase inhibitor, it is rational that the response to erlotinib may be related to the expression level of EGFR and activation of downstream signaling in PC cells. Therefore, we investigated the mRNA and protein expression levels of ARNTL2 and EGFR and activation of downstream signaling in the normal pancreatic duct (HPNE) and PC cell lines (BxPC-3, CFPAC-1, AsPC-1, PANC-1, Capan-1, PATU-8988 T and MIAPaCa-2). The ARNTL2 mRNA and protein expression levels were elevated in PC cells compared with normal pancreas cells. ARNTL2 was mainly up-regulated in CFPAC-1, AsPC-1, and PANC-1 cells at both mRNA and protein levels (Fig. 3F). Based on these results, HPNE, BxPC-3, CFPAC-1, AsPC-1, PANC-1, and PATU-8988 T cells were selected for subsequent experiments to represent PDAC cells with normal, high, and low levels of ARNTL2 expression, respectively. While a significant positive correlation between EGFR and ARNTL2 is not evident, it is clear that the PI3K/AKT signaling pathway, a downstream component of EGFR signaling, is notably activated in pancreatic cancer (PC) cell lines exhibiting high ARNTL2 expression (Fig. 3G), suggesting an important role of ARNNTL2 in regulating EGFR signaling.

### *PC cells with high ARNTL2 expression display sensitivity to erlotinib *in vitro

**Fig. 4:**
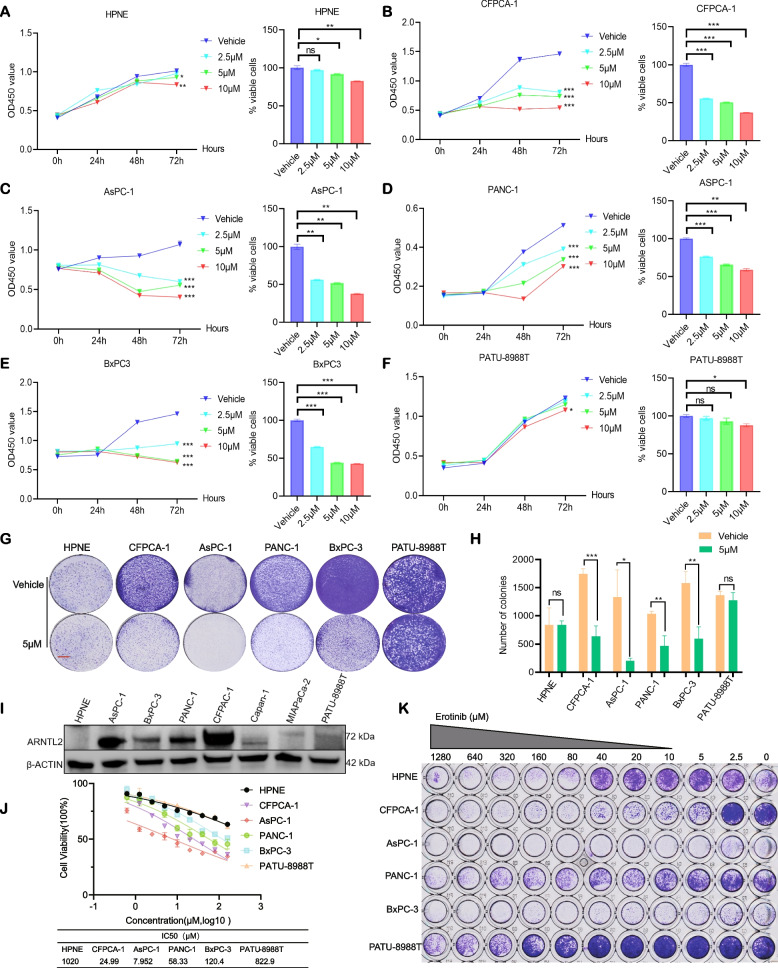
ARNTL2 highly expressed PC cells are sensitive to erlotinib in vitro. Cell proliferation of HPNE cells (**A**), CFPAC-1 (**B**), AsPC-1 (**C**), PANC-1 (**D**), BxPC-3 (**E**) and PATU-8988 T (**F**) treated for 72 h with erlotinib at 2.5 μM, 5 μM and 10 μM, respectively; **G**-H Colony-formation assay of 5 pancreatic cancer cell lines and one normal pancreatic duct cells were grown in the absence or presence of erlotinib at the indicated concentrations for 7–10 days, fixed and stained; **I** ARNTL2 expression levels across the PC cell lines; **J** IC50 assay of erlotinib in pancreatic cancer cell lines and normal pancreatic duct cells (K) Synergistic response to erlotinib treatment in pancreatic cancer cell lines and normal pancreatic duct cells. All data are presented as the mean ± SEM of triplicate experiments. *p < 0.05; **p < 0.01; ***p < 0.001 by repeated measures with Student’s t test

To investigate whether ARNTL2 serves as a biomarker of erlotinib response, we examined the impact of ARNTL2 expression on tumor cell proliferation in the presence or absence of erlotinib. Cell lines (HPNE, BxPC-3, CFPAC-1, AsPC-1, PANC-1, and PATU-8988 T) were incubated with increasing concentrations of erlotinib (2.5 μM, 5 μM, and 10 μM) for different incubation periods (24, 48, and 72 h). Treatment with erlotinib (10 μM) for 72 h reduced cell proliferation by 70%-40% in CFPAC-1, AsPC-1, PANC-1, and BxPC-3 cells (Fig. 4B-E). Incubation with erlotinib (10 μM) decreased the proliferation of HPNE and PATU-8988 T by 10% and by 20% (Fig. 4A, F). Next, we used colony-formation assay and IC50 assay to evaluate the sensitivity of PC cell lines to erlotinib. These experiments revealed that PC cell lines with high ARNTL2 expression (BxPC-3, CFPAC-1, AsPC-1, and PANC-1 cells) were more sensitive to erlotinib compared with control cells, whereas HPNE and PATU-8988 T negative for ARNTL2 were not sensitive to erlotinib (Fig. 4G-K).

### ARNTL2 knockdown limits the response of PC cells to erlotinib

**Fig. 5:**
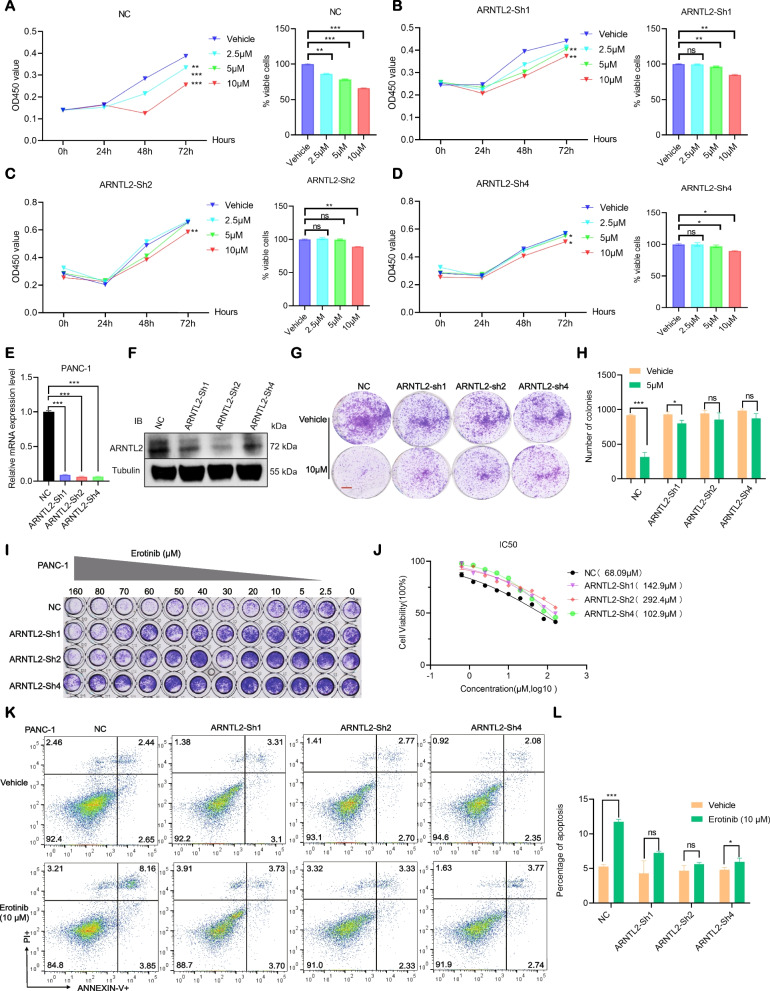
ARNTL2 knockdown limits the response of PC cells to erlotinib. **A**-**D** Cell proliferation of PANC-1 cells incubated with shRNA-1, shRNA-2, and shRNA-4 against ARNTL2 or control (NC) and treated for 72 h with erlotinib at 2.5 μM, 5 μM, and 10 μM, respectively; **E**–**F** ARNTL2 knockdown efficiency of the shRNAs was measured by western blotting and q-PCR, respectively; **G**-**H** Colony-formation assay of PANC-1 cells incubated with shRNA-1, shRNA-2 and shRNA-4 against ARNTL2 or control (NC) were grown in the absence or presence of erlotinib at the indicated concentrations for 7–10 days, fixed and stained; **I** Synergistic response to erlotinib treatment in PANC-1 cells incubated with shRNA-1, shRNA-2 and shRNA-4 against ARNTL2 or control (NC); **J** IC50 assay of erlotinib in PANC-1 cells incubated with shRNA-1, shRNA-2 and shRNA-4 against ARNTL2 or control (NC). **K**-**L** Flow cytometry analysis of erlotinib-induced cell apoptosis in ARNTL2 knockdown PANC-1 cells treated with erlotinib and stained with Annexin V-APC-633/PI. All data are presented as the mean ± SEM of triplicate experiments. *p < 0.05; **p < 0.01; ***p < 0.001 by repeated measures with Student’s t test

In support of the notion that PC cells with high ARNTL2 expression are sensitive to erlotinib, we showed that three different shRNA directed against ARNTL2 significantly reduced PANC-1 cell sensitivity to erlotinib when compared with vehicle cells (Fig. 5E-F). In addition, the Cck8 assay confirmed that ARNTL2 knockdown minimized the inhibitory effect of erlotinib on PANC-1 cell proliferation (Fig. 5A-D). We also found that ARNTL2 knockdown reduced PANC-1 cell sensitivity to erlotinib when compared with vehicle cells in colony formation and IC50 assays (Fig. 5G-J). Furthermore, flow cytometry performed to evaluate between early apoptotic (Annexin V^+^PI^−^) and late apoptotic or necrotic (Annexin V^+^PI^+^) cells, we found that ARNTL2 knockdown in PANC-1 cells significantly suppressed erlotinib-induced cell apoptosis and necrosis than that in the control group (Fig. 5K-L). These results indicated that ARNTL2 knockdown limits the response of PC cells to erlotinib treatment via reducing erlotinib-induced cell apoptosis and necrosis.

### Overexpression of ARNTL2 sensitizes PC cells to erlotinib

**Fig. 6:**
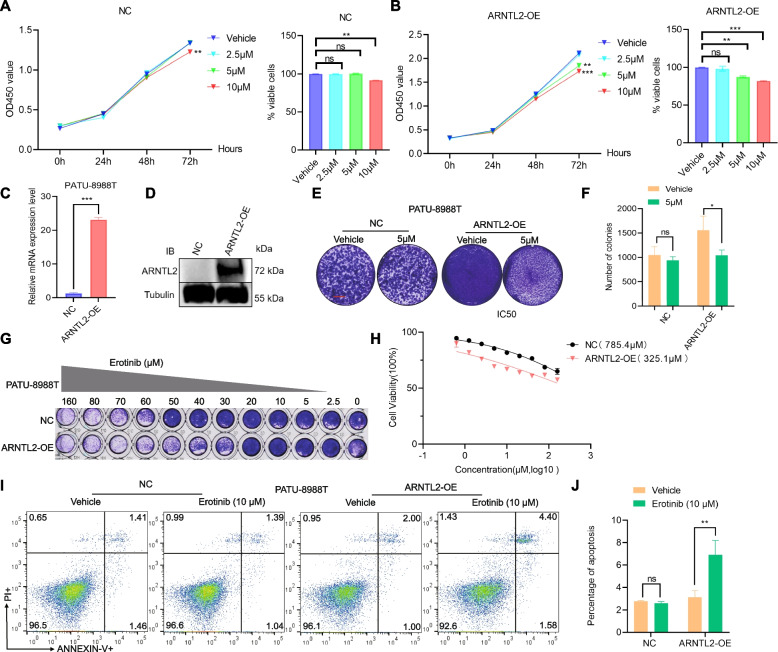
Overexpression of ARNTL2 sensitizes PC cells to erlotinib treatment. **A**-**B** Cell proliferation of ARNTL-vector, ARNTL2-OE PATU-8988 T and treated for 72 h with erlotinib at 2.5 μM, 5 μM and 10 μM, respectively; **C**-**D** ARNTL2 overexpression efficiency was measured by western blotting and q-PCR, respectively; **E**–**F** Colony-formation assay of ARNTL2-overexpression PATU-8988 T cells and control cells(NC) were grown in the absence or presence of erlotinib at the indicated concentrations for 7–10 days, fixed and stained; **G** Synergistic response to erlotinib treatment in ARNTL2-overexpression PATU-8988 T cells and control cells(NC); **H** IC50 assay of erlotinib in ARNTL2-overexpression PATU-8988 T cells and control cells(NC); **I**-**J** Flow cytometry analysis of erlotinib-induced cell apoptosis in ARNTL2-overexpression PATU-8988 T cells and control cells(NC) treated with erlotinib and stained with Annexin V-APC-633/PI. All data are presented as the mean ± SEM of triplicate experiments. *p < 0.05; **p < 0.01; ***p < 0.001 by repeated measures with Student’s t test

To complement knockdown experiments, we overexpressed ARNTL2 in PATU-8988 T cells (Fig. 6C-D), which naturally expressed very low levels of ARNTL2. Subsequent treatment with erlotinib led to a 10% reduction in cell proliferation in PATU-8988 T cells, while control cells exhibited a more pronounced 30% decrease. (Fig. 6A-B). In agreement with these data, colony-formation assay and IC50 assay confirmed that overexpression of ARNTL2 substantially sensitized PATU-8988 T cells to erlotinib treatment (Fig. 6E-H). Finally, in erlotinib treated cells flow cytometry revealed a significantly higher proportion of apoptosis in cells overexpressing ARNTL2 than that in the control group (Fig. 6I-J). Taken together, these findings underscore the in vitro sensitivity of pancreatic cancer cells with elevated ARNTL2 expression to erlotinib.

### Patient-derived xenografts suggest erlotinib therapeutic potential for patients with high ARNTL2 expression

**Fig. 7:**
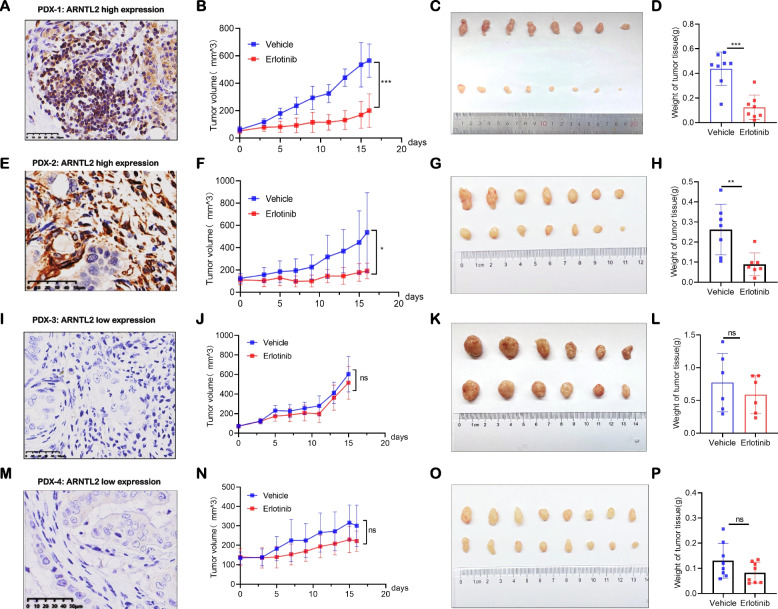
Erlotinib has strong therapeutic implications for ARNTL2-high patients in patient-derived xenografts. **A**, **E**, **I**, **M** Images illustrating the IHC staining for human ARNTL2 in PC-PDX1 (**A**), PC-PDX2 (**E**), PC-PDX3(I), and PC-PDX4(M); (**B**, **F**, **J**, **N**) Tumor growth curves of PC-PDX1 (**B**), PC-PDX2 (**F**), PC-PDX3 (**J**) and PC-PDX4 (**N**) treated with erlotinib and vehicle. Arrows indicate the day when treatment started; (**C**, **G**, **K**, **O**) Representative tumor images of erlotinib and vehicle group of PC-PDX1 (**C**), PC-PDX2 (**G**), PC-PDX3(K) and PC-PDX4 (**O**) at the end of treatment; (**D**, **H**, **L**, **P**) Quantitative analysis of PC-PDX1 (**D**), PC-PDX2(H), PC-PDX3 (**L**) and PC-PDX4(P) weight at the end of treatment; All data are presented as the mean ± SEM of triplicate experiments. **p < 0.01; ***p < 0.001 by repeated measures with Student’s t-test

We next examined the impact of ARNTL2 expression in vivo tumor response to erlotinib treatment using 4 PC patient-derived xenograft (PDX) mouse models. The mice received a daily oral erlotinib treatment 100 mg/kg. Using an immunohistochemistry score of ARNTL2, we selected PC-PDX-1 and PC-PDX-2 expressing a high level of ARNTL2 (Fig. 7A, E), whereas PC-PDX-3 and PC-PDX-4 with ARNTL2 were characterized with a loss or low expression (Fig. 7I, M). We then compared the effects of erlotinib treatment on the four PC-PDX models expressing various levels of ARNTL2. Our results showed that ARNTL2-high PC-PDX-1 and PC-PDX-2 were more sensitive to erlotinib treatment and showed a substantial reduction in vivo tumor growth (Fig. 7B-D and Fig. 7F-H). Nonetheless, PC-PDX-3 and PC-PDX-4, characterized by ARNTL2 loss or low expression, demonstrated restricted effectiveness of erlotinib in inhibiting tumor growth (Fig. 7J-L and Fig. 7N-P). As expected, IHC staining of ARNTL2, EGFR, and the proliferation marker Ki67 were significantly decreased in the erlotinib treatment group (Figure S3). In line with the in vitro results, ARNTL2 sensitized tumors to erlotinib as a single agent, highlighting ARNTL2 as a predictive biomarker for erlotinib response in pancreatic cancer.

### *ARNTL2/E2F1 axis-mediated glycolysis sensitizes PC cells to erlotinib treatment *via* activating PI3K/AKT signaling pathway*

**Fig. 8:**
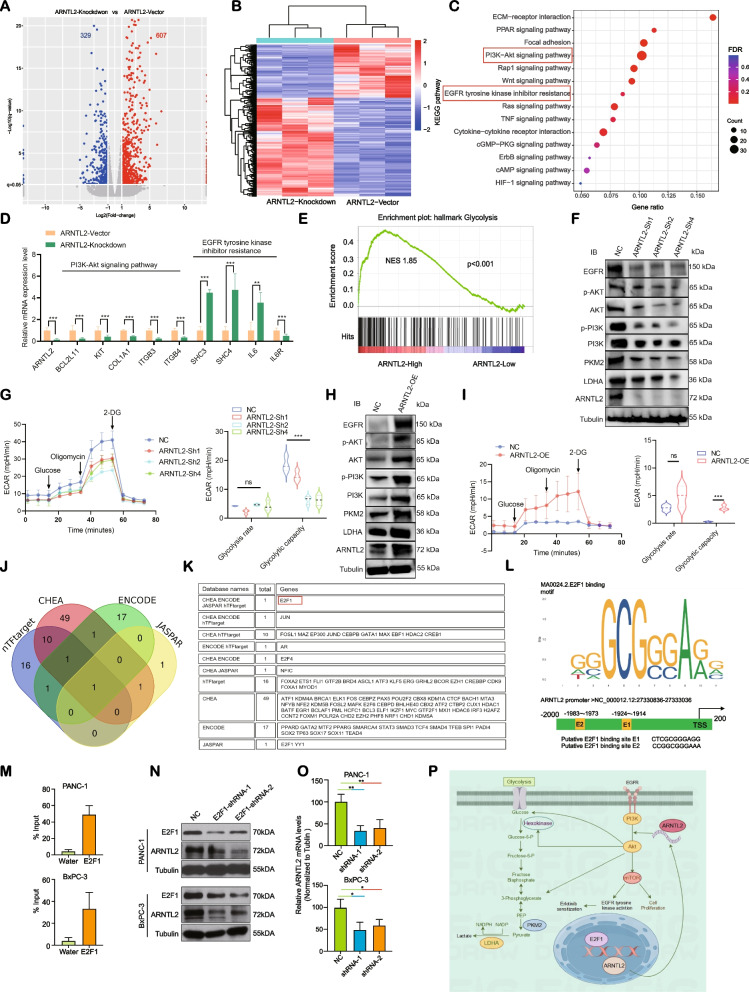
ARNTL2/E2F1 axis-mediated glycolysis sensitizes PC cells to erlotinib treatment via activating the PI3K/AKT pathway. **A** Volcano plot of differentially expressed gene profiles (ARNTL2-Knockdown vs. ARNTL2-Vector); **B** Heatmap of the indicated genes in PANC-1 cells with or without ARNTL2 knockdown; **C** KEGG analysis indicated that PI3K-Akt signaling pathway served as one of the major enriched signaling in ARNTL2-Knockdown group; **D** The mRNA levels of the indicated genes in PANC-1 cells with or without ARNTL2 knockdown; **E** GSEA analysis identified the glycolytic status in high ARNTL2 expression group; **F** Western blot analysis identified that the protein levels of PI3K-Akt signaling pathway and glycolysis related molecules in PANC-1 cells were decreased in ARNTL2-silenced group; **G** Glycolysis rate of PANC-1 cells ARNTL2 knockdown was examined by a Seahorse XFe96 Glycolysis Stress Test analyzer; **H** Western blot analysis identified that the protein levels of PI3K-Akt signaling pathway and glycolysis related molecules in PATU-8988 T cells were increased in ARNTL2-overexpressed group; **I** Glycolysis rate of PATU-8988 T cells overexpressing ARNTL2 was examined by a Seahorse XFe96 Glycolysis Stress Test analyzer; **J**-**K** Venn diagrams of four gene lists: transcription factors predicted by the hTFtarget, CHEA, ENCODE and JASPAR databases, ARNTL2 is transcriptionally activated by E2F1; **L** E2F1-binding motifs and predicted E2F1-binding sites (E1and E2) on the promoter region of ARNTL2 were obtained from the JASPAR database; **M** Chromatins were isolated from PANC-1 and BxPC-3 cells. The binding of E2F1 and blank control (Water) to the ARNTL2 promoter was tested using ChIP assay. **N** and **O** PANC-1 and BxPC-3 cells were incubated with E2F1 shRNA-1 and shRNA-2 or control (NC), western blot and qRT-PCR were used to test the protein and mRNA levels of ARNTL2. **P** Schematic representation depicting the mechanisms that the E2F1/ARNTL2 mediated glycolysis sensitizes PC cells to erlotinib treatment via activating PI3K/AKT pathway. All data are presented as the mean ± SEM of triplicate experiments. *p < 0.05, **p < 0.01 by repeated measures with Student’s t-test

To determine the underlying mechanisms of ARNTL2 mediating glycolysis and the responsiveness of PC cells to erlotinib we performed RNA-seq of PANC-1 cells following ARNTL2-Knockdown or expression of an ARNTL2-Vector. The analysis of differentially expressed genes (DEGs) showed significant differences in gene expression profiles (Fig. 8A-B). Analyses of the KEGG pathway based on these DEGs, revealed that the PI3K/AKT signaling pathway was an overwhelmingly enriched in ARNTL2 high-expression PC cells (Fig. 8C). In agreement with these data, ARNTL2 depletion was associated with a reduced expression of genes involved in the PI3K/AKT signaling pathway. In addition, the knockdown of ARNTL2 was associated with an increased expression of genes in the EGFR TKI resistance pathway (Fig. 8D). Since PI3K/Akt signaling pathway is closely associated with cancer proliferation and glucose metabolism [28], we next performed a GSEA analysis and confirmed the enrichment of glycolysis in tissues with high ARNTL2 expression (Fig. 8E). Moreover, the protein expression levels of genes in the PI3K/AKT pathway were induced by ARNTL2 knockdown (Fig. 8F). To further study the interaction between ARNTL2 level and glycolysis, we analyzed the real-time glycolytic rate in both gain-of-function and loss-of-function experimental systems by knocking down ARNTL2 in PANC-1 cells and by overexpressing ARNTL2 in PATU-8988 T. Our studies demonstrated that ARNTL2 knockdown in PANC-1 significantly suppressed the glycolytic capacity, while the overexpression of ARNTL2 in PATU-8988 T had the opposite effect (Fig. 8G-H). In line with these results, the overexpression of ARNTL2 enhanced the glycolytic capacity (Fig. 8I). These results revealed that ARNTL2 facilitates cellular glycolytic capacity and erlotinib response for patients with ARNTL2 high expression.

To predict potential transcription factors (TFs) that could regulate ARNTL2 expression, we further performed analyses of the hTFtarget, CHEA, ENCODE, and JASPAR databases. As displayed in Fig. 8 J-K, E2F1 was identified as the upstream transcription factor of ARNTL2. To further verify whether E2F1 regulates ARNTL2 expression, we first analyzed the presence of potential E2F1 binding site in the ARNTL2 promoter sequence (Fig. 8L). Then ChIP assay was conducted. An anti-E2F1 antibody amplified a 171-bp DNA fragment of the ARNTL2 promoter (Fig. 8M), suggesting that E2F1 directly bound to the ARNTL2 promoter. We then confirmed the regulatory effect of E2F1 on ARNTL2 expression. We found that both protein and mRNA levels of ARNTL2 were decreased in E2F1 knockdown groups (Fig. 8N and O).

Our studies suggest that in fact E2F1 could bind to the promoter region of the ARNTL2 gene locus, thereby leading to the activation of the PI3K/AKT signaling pathway in PC cells (Fig. 8P).

## Discussion

Pancreatic cancer is an aggressive malignancy with an unfavorable prognosis, limited therapeutic options, and low efficacy of current treatment due to early metastases and recurrences, resulting in less than a 10% 5-year overall survival rate [4, 29]. Despite advancements in new diagnostic methods and therapeutic strategies, there has been limited translation of novel findings into clinical benefits. The high heterogeneity of PC tumors makes it challenging to find a therapy with broad application. Therefore, design of tailored treatment strategies for specific patients’ groups, a main objective of this study, holds great significance in maximizing therapeutic effects. In this study, we identified ARNTL2 as a prognostic biomarker and sensitivity predictor for erlotinib in PC patients. Additionally, we developed a glycolysis-related gene signature (GRS) based on the expression of two biomarkers (ARNTL2 and TPX2), which serves as a prognostic signature and effectively discriminates high-risk patients in PC. Although the two biomarkers included in our gene signature have been studied in multiple cancers, their investigation in tumor glycolysis as well as their role as biomarkers for guiding targeted treatment has been limited [30, 31].

ARNTL2 serves as an important transcriptional activator and is a core component of the circadian clock, playing a role in regulating cell proliferation, migration, and invasion [15]. A recent study demonstrated ARNTL2's oncogenic properties and found a strong association between high levels of ARNTL2 and aggressive malignant phenotypes and poor survival in PC patients [20]. Our work built on these findings through a series of in vivo and in vitro experiments, showing that ARNTL2 is highly expressed in PCs and that its expression levels are inversely correlated with overall survival (OS) and disease-free survival (DFS) in pancreatic cancer patients. Furthermore, the expression levels of ARNTL2 showed a linear increase with higher GRS scores. TPX2 (Xenopus kinesin-like protein 2) has also been found to be upregulated in various tumors, including gastric cancer, colorectal carcinoma, hepatocellular carcinoma, and bladder cancer [32–35]. Numerous studies have reported the tumorigenic and prognostic role of TPX2 in various cancers [36–38]. Given that ARNTL2 is a significant factor for the GRS, it is likely a suitable biomarker to predict the prognosis and curative effect of high-GRS pancreatic cancer patients. Thus, we choose ARNTL2 as the core marker gene of GRS for our study. We used a panel of in vitro assays and in vivo validation in PDX-PC models and demonstrated that ARNTL2 is an excellent biomarker for predicting the prognosis and therapeutic effect of erlotinib treatment in PC. Considering that ARNTL2 is a significant factor for the GRS, we thought it would also be a suitable biomarker for predicting prognosis and therapeutic effects in high-GRS pancreatic cancer patients. To test this hypothesis, we selected ARNTL2 for further study on the GRS. PDX model is an ideal model which capture the tumor characteristics and stromal environment of PC [39, 40]. Using a series of in vitro assays and in vivo validation in PDX-PC models, we confirmed ARNTL2 is a reliable biomarker for predicting prognosis and therapeutic effects of erlotinib treatment in PC.

Previous studies have demonstrated that EGFR is mostly upregulated in pancreatic tumors and is associated with a worse prognosis [41]. Deregulated EGFR signaling has also been implicated in the development and malignant progression of PC [42, 43]. Currently, anti-EGFR-targeted therapies are ineffective in unselected PC patients, leading to limited success [44]. Also, the lack of well-recognized feasible targets in PC sub-classification and reliable predictors for prognosis hinders the use of available targeted therapies. Erlotinib is an FDA-approved oral HER1/EGFR tyrosine kinase inhibitor that blocks tumor cell division, produces cell cycle arrest, and initiates programmed cell death in EGFR-overexpressing human tumor cells [45]. However an obvious clinical benefit of erlotinib on survival was not seen when used with gemcitabine in patients with advanced pancreatic cancer [46]. As a result, application of erlotinib in advanced pancreatic cancer is still limited. Many studies have revealed that specific factors played key roles in cancer therapeutic resistance and also could be the biomarkers [47–49]. In our study, we demonstrate that anti-EGFR is effective in PC patients with higher expression of ARNTL2 as demonstrated in our studies using various pancreatic cancer cell lines and PC-PDX models. Importantly, we found that cells with high expression of ARNTL2 are strongly sensitive to erlotinib therapy and, for the first time to our knowledge, confirmed these observations in xenograft models. Our studies support the notion that ARNTL2 can be used as a triage marker to determine subgroups that can benefit from erlotinib treatment. In fact, we describe two subgroups of PC with distinct responses to erlotinib: sensitive tumors (with a high ARNTL2 expression) and insensitive tumors (with a low ARNTL2 expression), independently of EGFR expression. Accordingly, in PC tissues expressing EGFR and which are nonresponsive to erlotinib treatment, ARNTL2 induction reversed tumor insensitivity and increased the antitumor effect of erlotinib. This indicates a potential clinical translation with induction of treatment sensitivity in PC patients’ resistance to EGFR inhibitors.

The role of EGFR signaling in regulating cellular metabolism including glycolysis has been extensively studied [50, 51]. Several studies have shown that EGFR enhanced glycolysis through PI3K/AKT activation [52, 53]. However, the role of ARNTL2 in regulating cellular glycolysis and drug sensitivity has not yet been studied. Our study uncover that ARNTL2 increases cellular glycolytic capacity and erlotinib response for patients with high ARNTL2 expression. In addition, ARNTL2 silencing suppressed the activation of the PI3K/AKT signaling. Moreover, our analyses of transcription factors revealed E2F1 could bind to the promoter region of ARNTL2, and up-regulate its expression. Numerous studies have proved that E2F1 was involved in regulating cell metabolism and proliferation via activating the PI3K/AKT signaling pathway [54]. These data uncover that the ARNTL2/E2F1 may serve as a novel predictive biomarker for choosing patients who may benefit from erlotinib therapy in PC. Thus, our work shows that patient stratification based on the expression of ARNTL2 could help to select the subgroup of PC patients who might benefit from EGFR inhibitors. This study has some limitations: (1) We defined ARNTL2 as a novel prognostic biomarker and predictive indicator of sensitivity. However, more direct evidence is needed to further confirm the mechanistic underpinnings of erlotinib sensitivity in pancreatic adenocarcinoma. (2) We demonstrated that ARNTL2 sensitized tumors to erlotinib as a single agent in the PDX model, but further validation in larger prospective trials and combined treatment with other chemotherapy drugs are necessary and warranted. Overall, our investigations have delineated ARNTL2 as a novel prognostic biomarker and predictive indicator of sensitivity, serving to identify erlotinib-responsive pancreatic cancer cases. These results contribute to advancing the field of precision oncology, enabling more accurate and targeted therapeutic interventions.

### Supplementary Information

**Supplementary file 1.**

**Supplementary file 2.**

## Data Availability

No datasets were generated or analysed during the current study.

## Abbreviations

ANOVA: One-way analysis of variance
AUC: Area under the curve
CCLs: Cancer cell lines
Cox-PH: Cox proportional-hazards
CTD2: Cancer Target Discovery and Development
GDSC: Genomics of Drug Sensitivity in Cancer
GRS: Glycolysis-related score
GTEx: Genotype-Tissue Expression Portal
HER1/ EGFR: Human epidermal growth factor receptor type1
MSigDB: Molecular Signatures Database
ICGC: International Cancer Genome Consortium portal
k-NN: K-nearest neighbor
LASSO: Least absolute shrinkage and selection operator
OS: Overall survival
PDX: Patient-derived xenografts
PC: Pancreatic adenocarcinoma
ssGSEA: Single-sample gene set enrichment analysis
TCGA: The Cancer Genome Atlas
tROC: Time-dependent receiver operating characteristic
WGCNA: Wweighted gene co-expression network analysis
