# Supplementary material for: Activation of the PI3K/AKT signaling pathway by ARNTL2 enhances cellular glycolysis and sensitizes pancreatic adenocarcinoma to erlotinib
Source: Mol Cancer. 2024 Mar 8;23:48. doi: 10.1186/s12943-024-01965-5 (PMC10921723; doi:10.1186/s12943-024-01965-5)
Supplement: Supplementary file 1 [file 12943_2024_1965_MOESM1_ESM.zip › Supplementry Figure legends.docx]

**Figure S1. Construction of a glycolysis-related gene signature.** (A) WGCNA was performed with whole-transcriptome profiling data and glycolysis ssGSEA Z-scores; (B) A total of 15 non-grey modules were identified, the magenta module exhibiting the highest relativity (r=0.37, *p*=8e−06) was deemed the most correlated with glycolysis; (C) Kaplan-Meier analysis of the OS between the low- and high-GRS groups; (D) The time-dependent ROC curves of the nomogram compared for 1-, 2-, and 3-year OS in PC, respectively.(E) Distribution of LASSO coefficients of the glycolysis-related gene signature. (F)Correlations between the expression of ARNTL2 and TPX2 and GRS score; (G) Nomogram for predicting the 1-, 2-, and 3-year OS of PC patients in the TCGA cohort.

**Figure S2. Evaluation of prognostic value of GRS.** (A-B) Kaplan-Meier curves of OS for patients stratified by median GRS in TCGA and ICGC cohorts; (C-D) Forrest plot of the univariate and multivariate Cox regression analyses in TCGA and ICGC cohorts; (E-F) The time-dependent ROC curves of the nomograms compared for GRS and other clinical characteristics in PC from TCGA and ICGC cohorts, respectively.

**Figure S3.** Representative H&E staining and Ki67, ARNTL2, EGFR IHC staining in xenografts from PC-PDXs after erlotinib treatment. Scale bars, 200 μm.
