# Supplementary figures and images for: Activation of the PI3K/AKT signaling pathway by ARNTL2 enhances cellular glycolysis and sensitizes pancreatic adenocarcinoma to erlotinib
Source: Mol Cancer. 2024 Mar 8;23:48. doi: 10.1186/s12943-024-01965-5 (PMC10921723; doi:10.1186/s12943-024-01965-5)

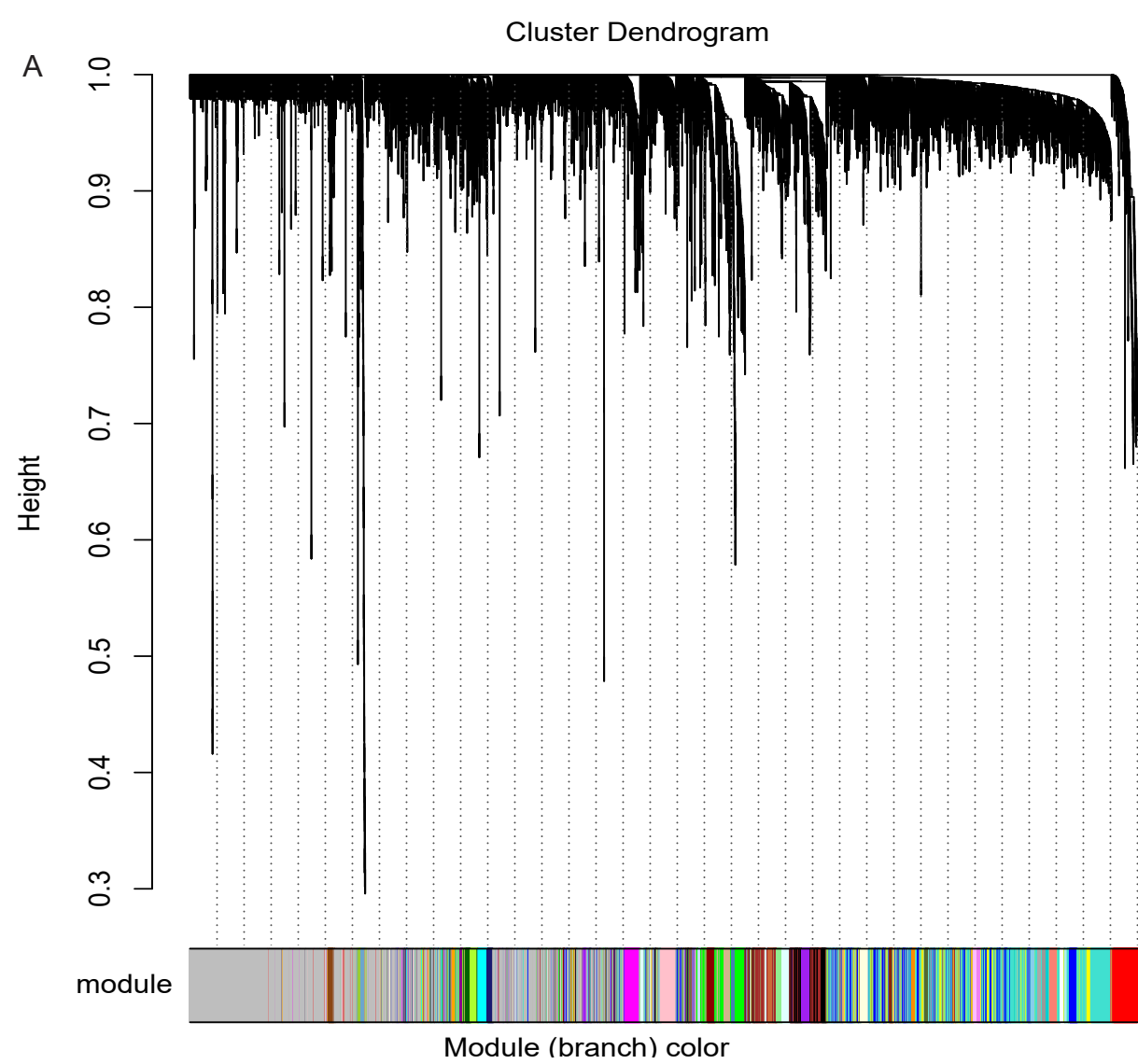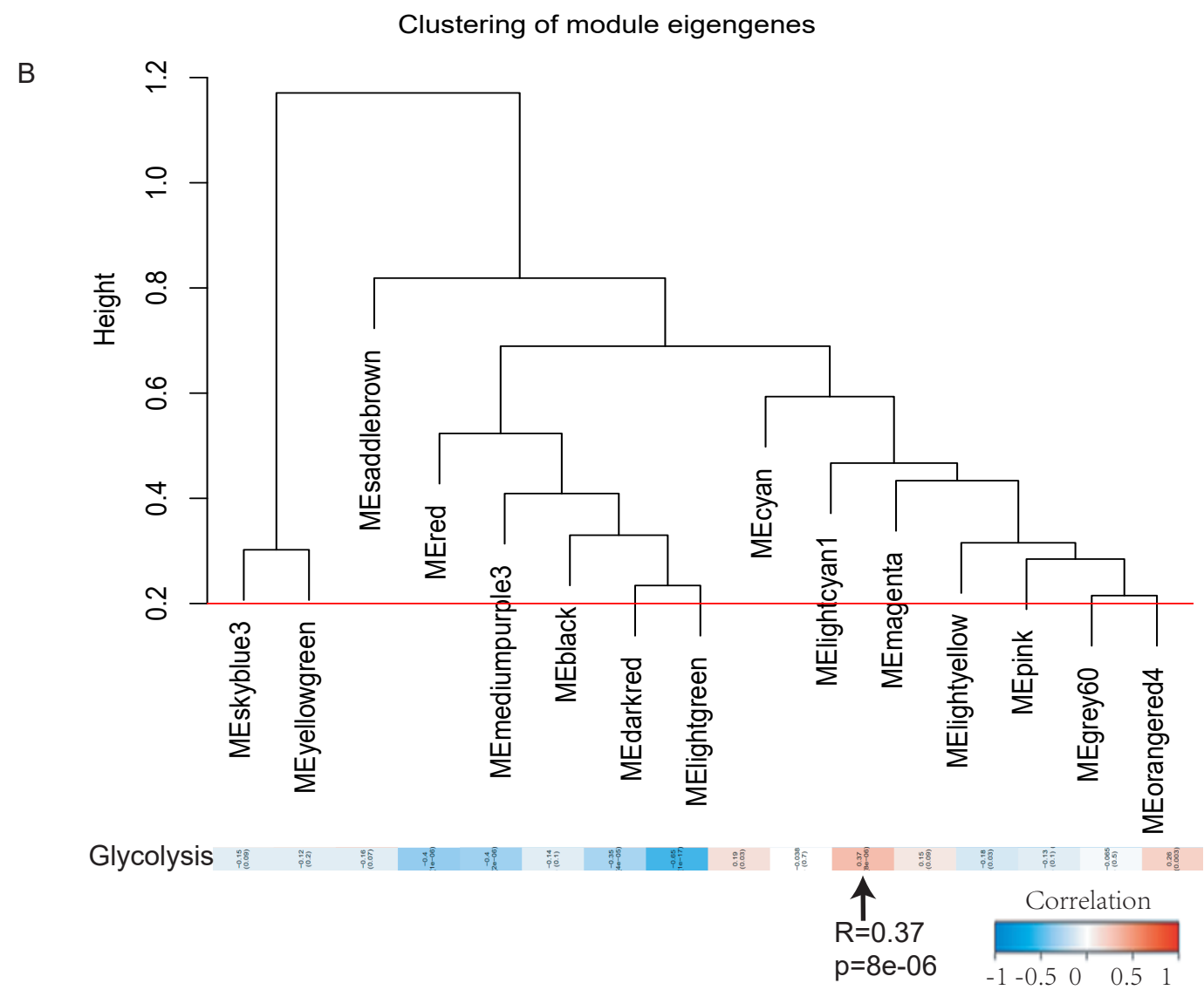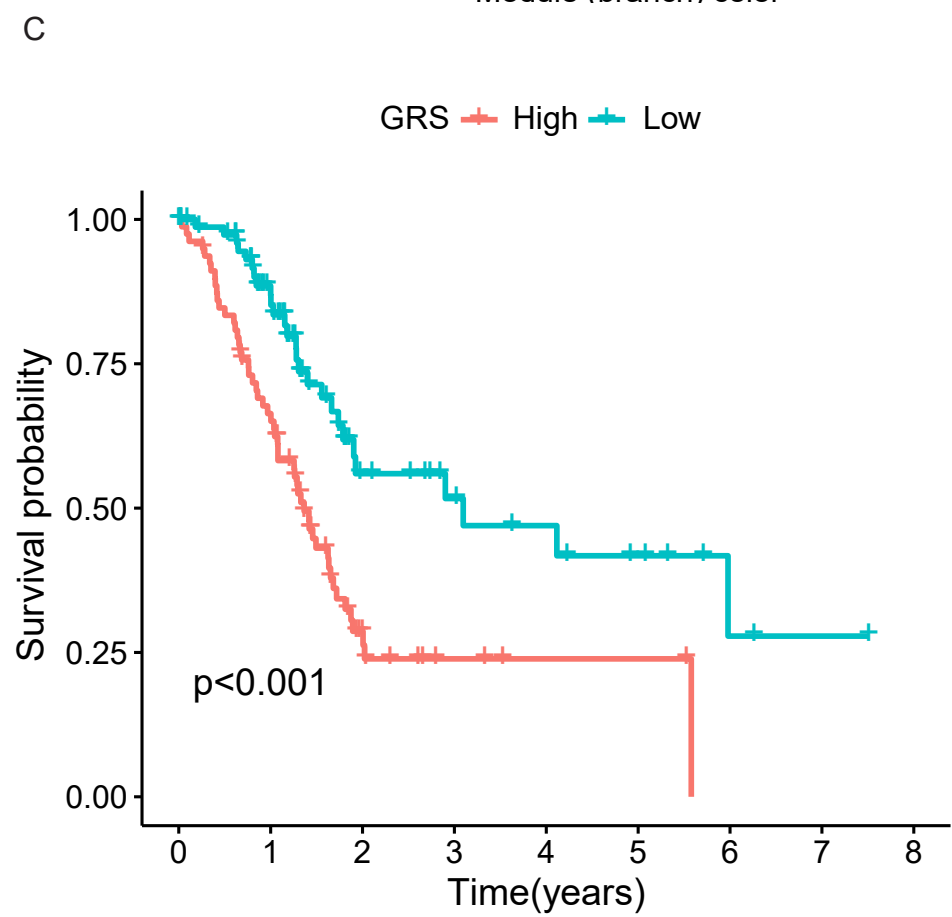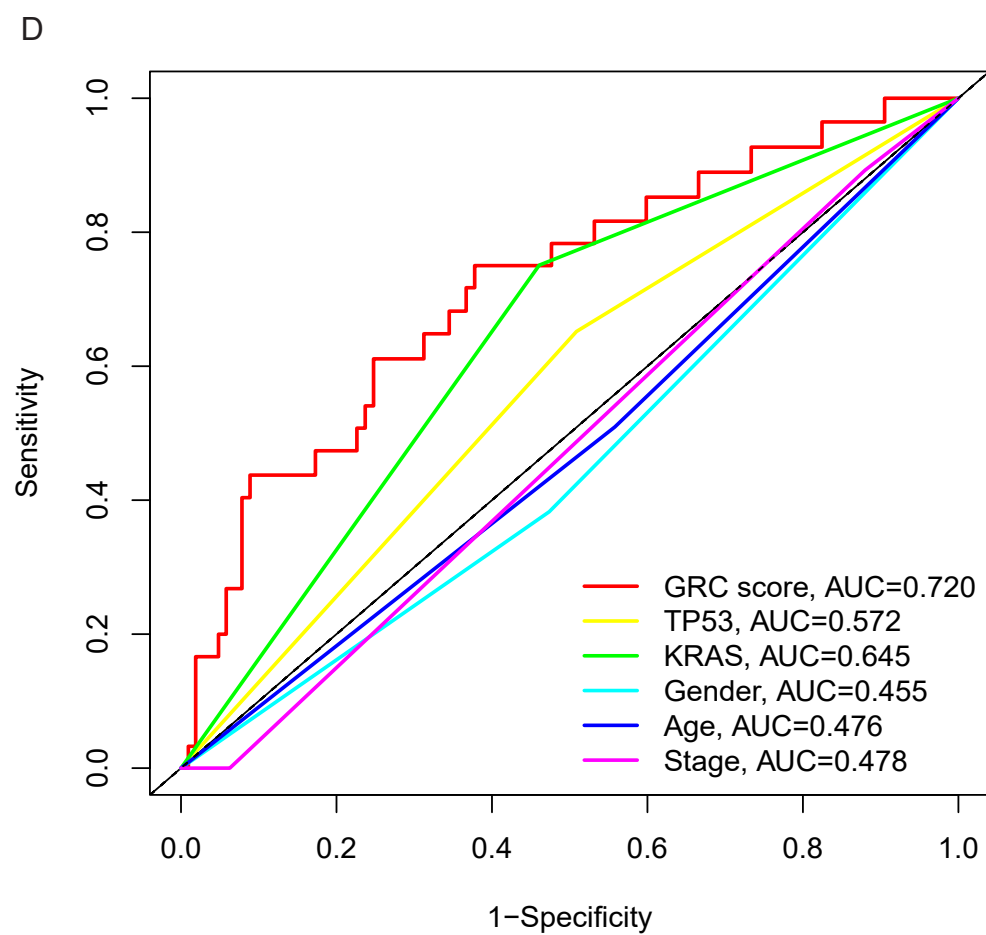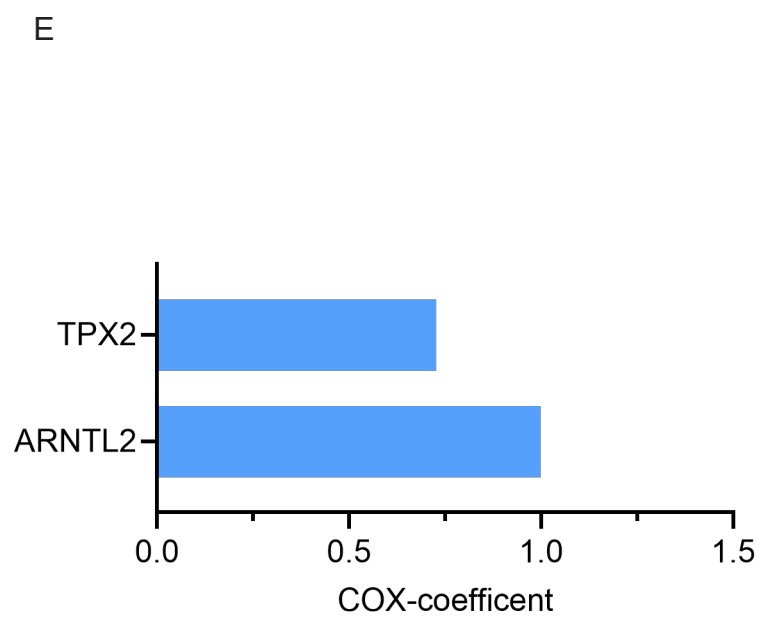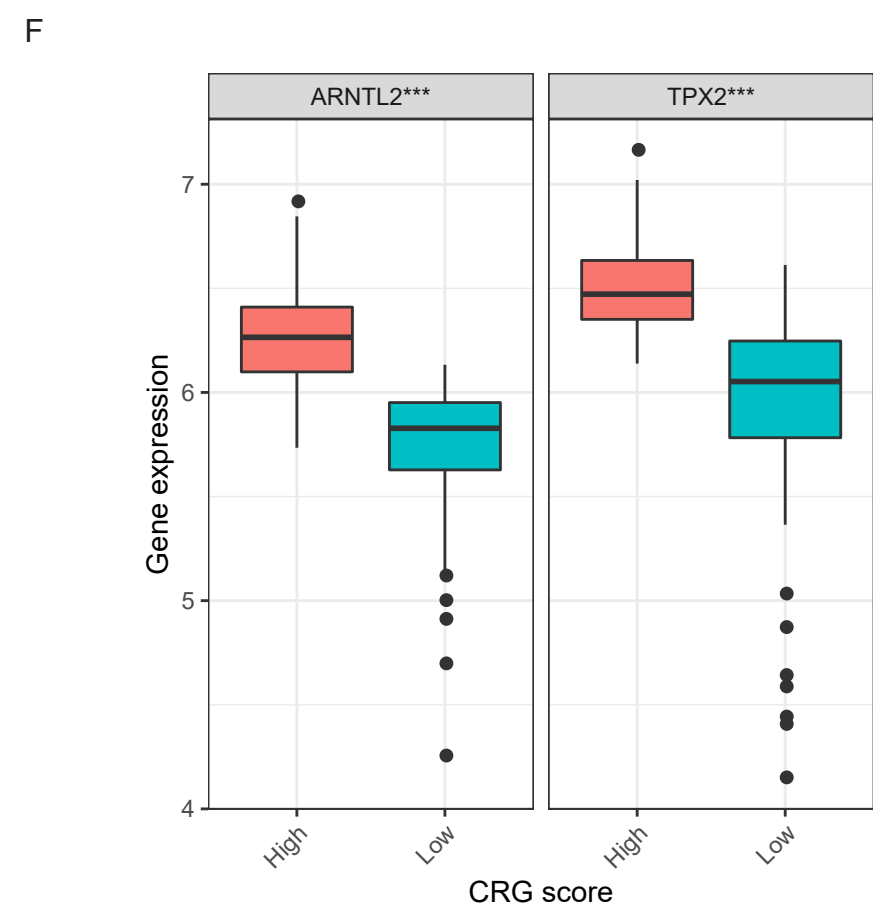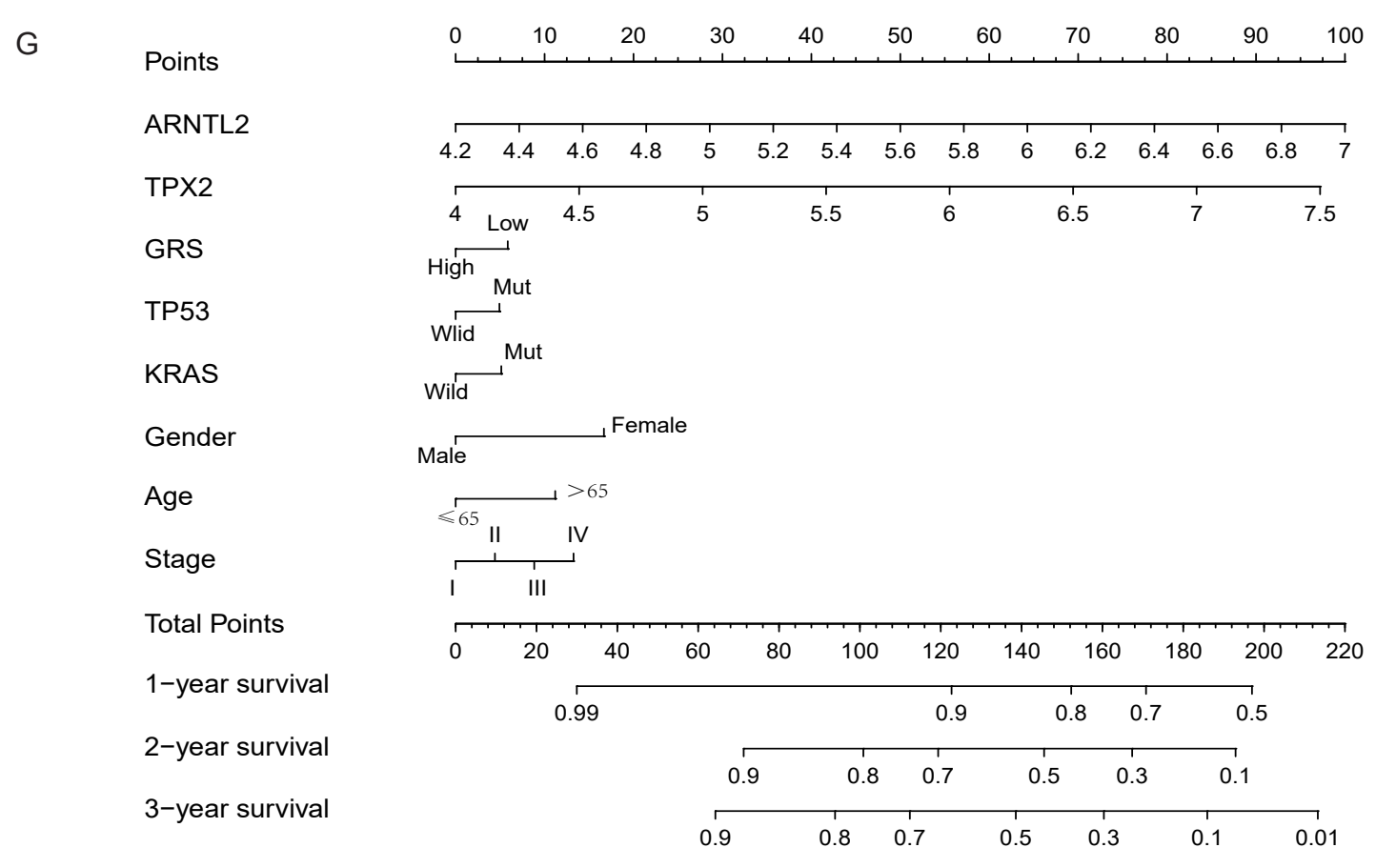

Supplement: Supplementary file 1 [file 12943_2024_1965_MOESM1_ESM.zip › S1.pdf]

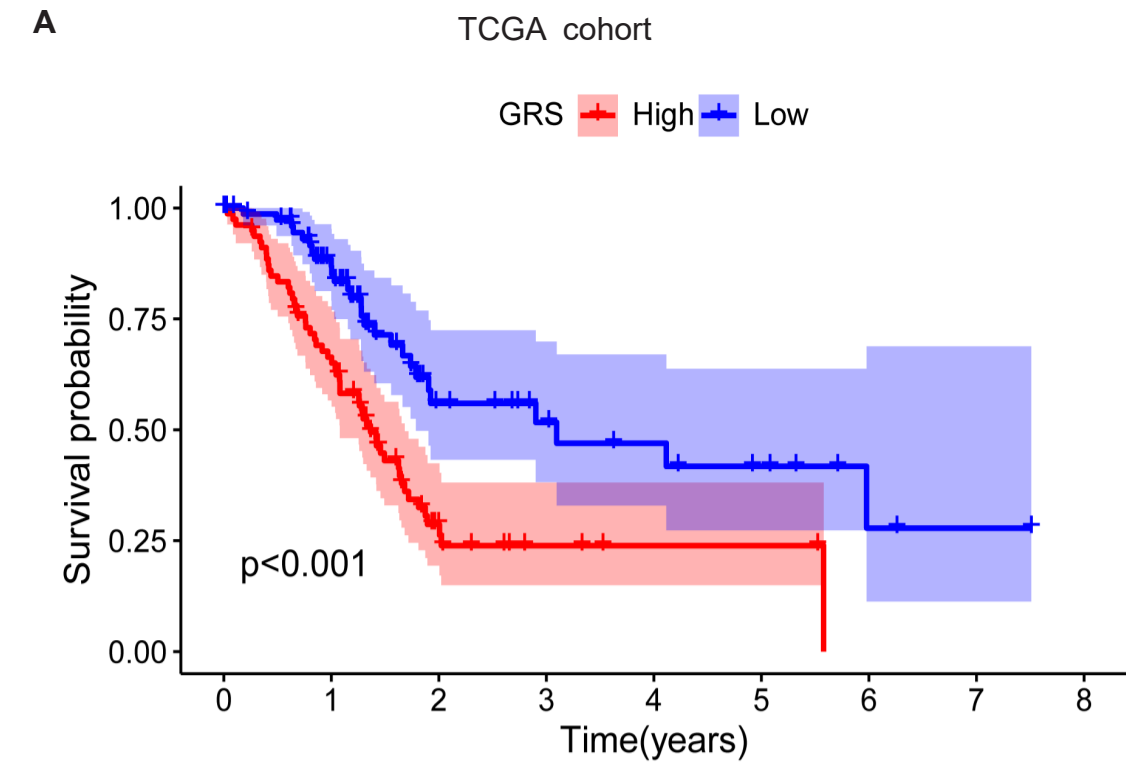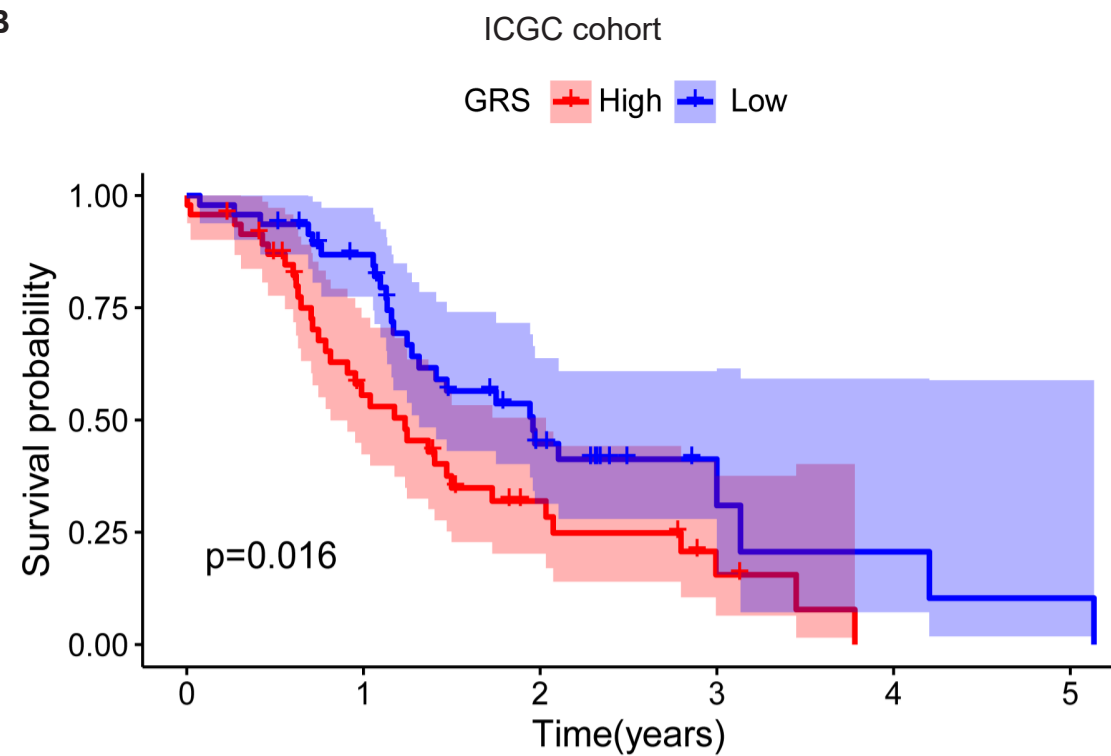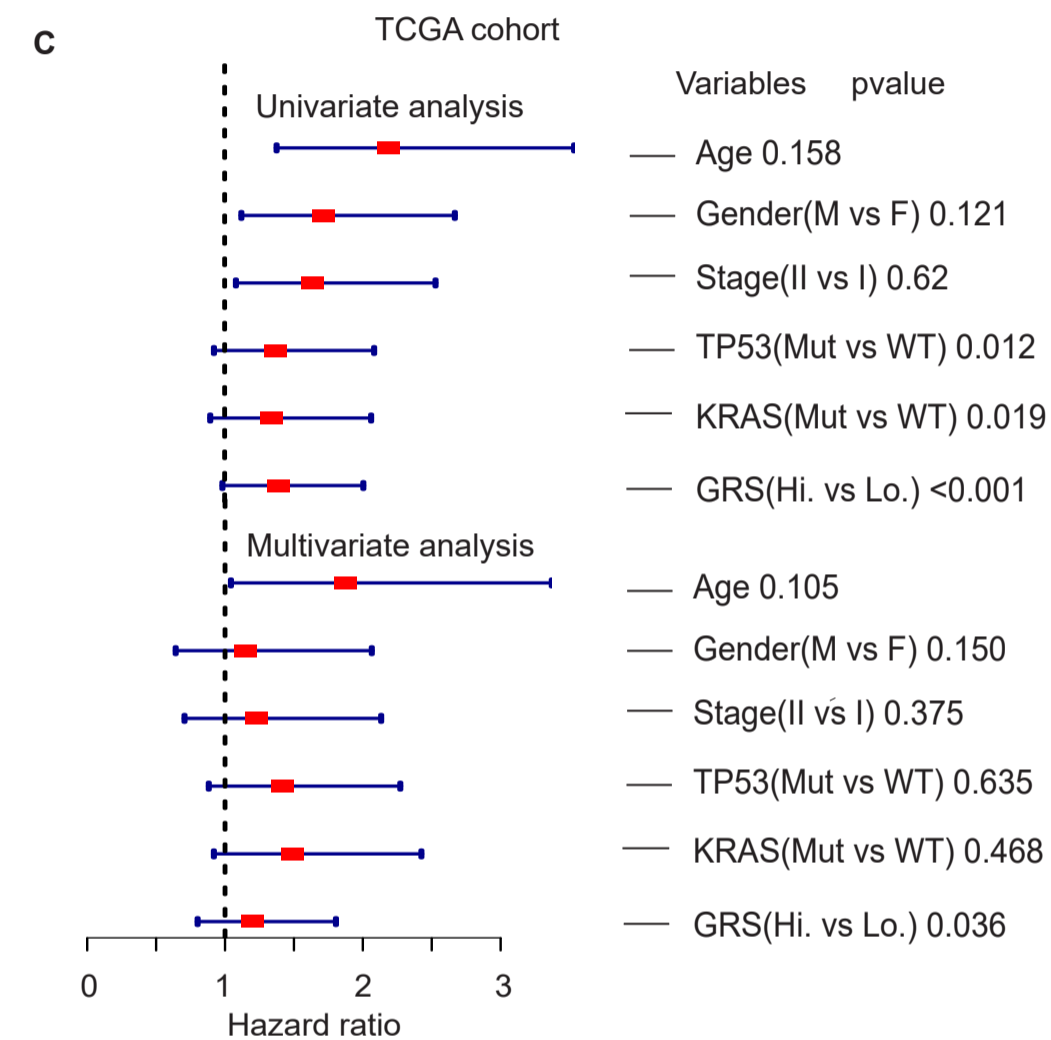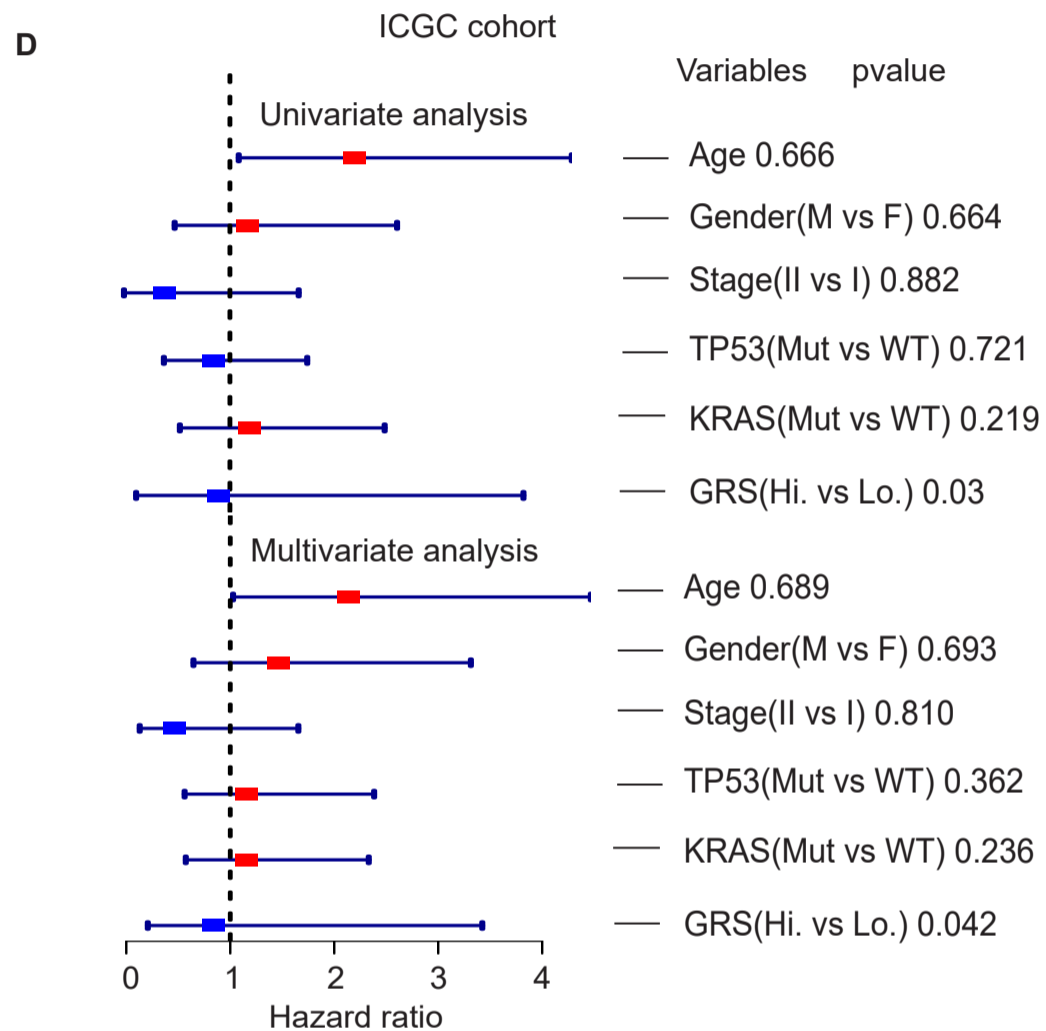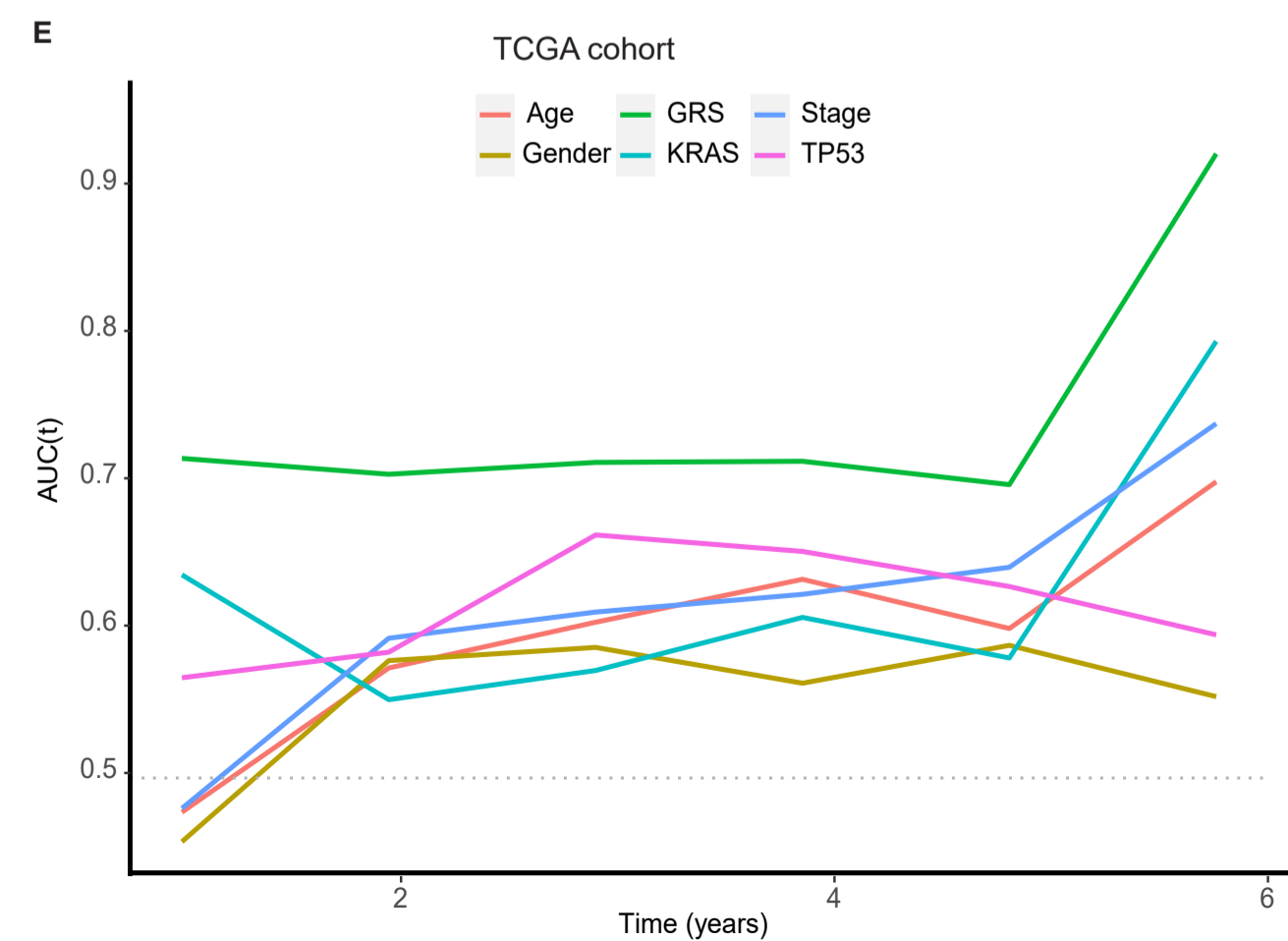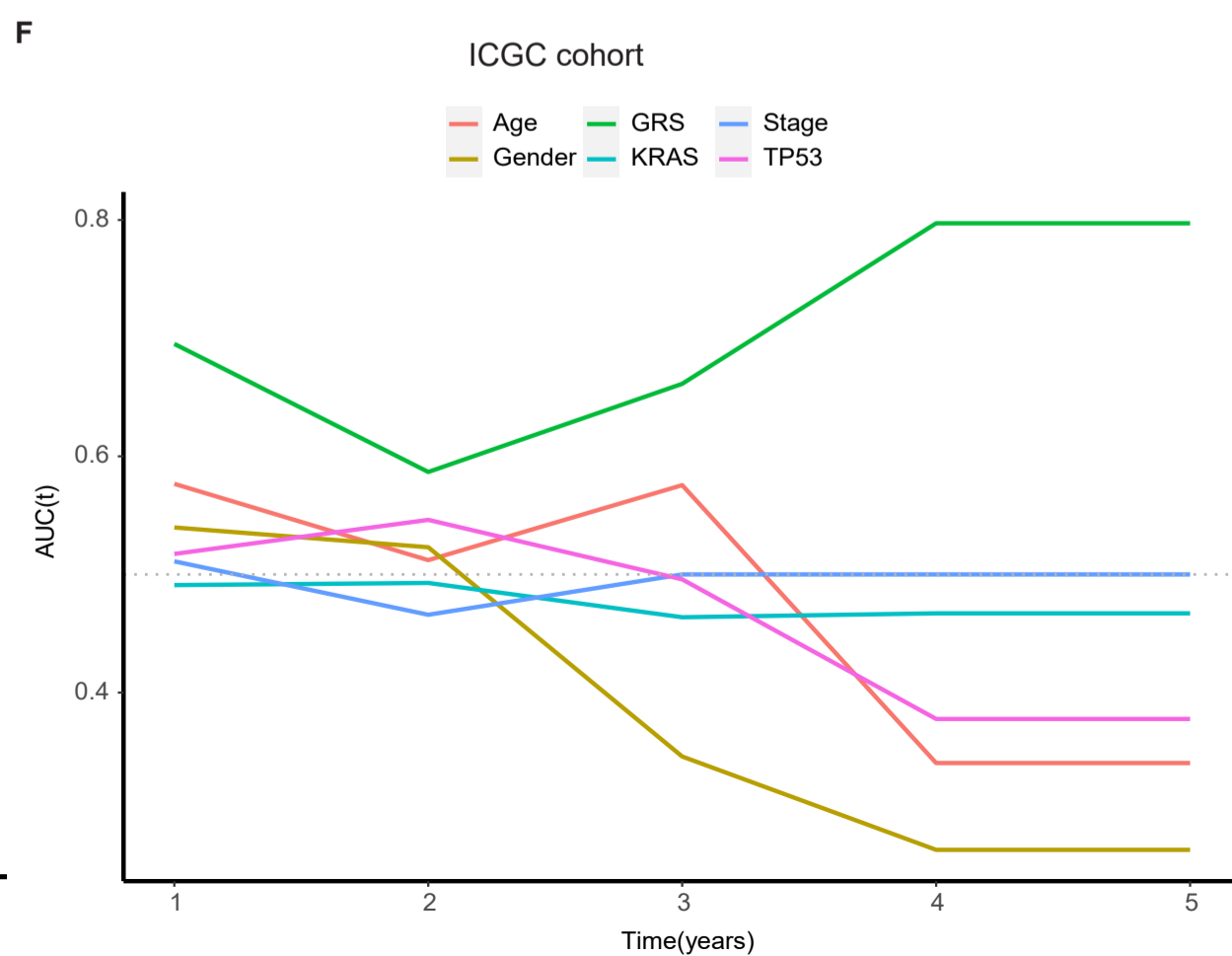

Supplement: Supplementary file 1 [file 12943_2024_1965_MOESM1_ESM.zip › S2.pdf]

**A****H&E**

Vehicle

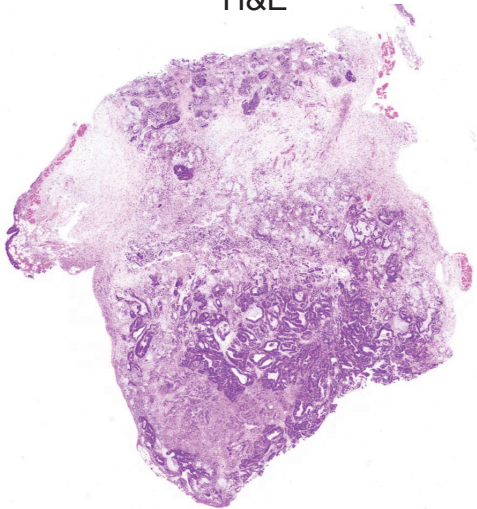

Erlotinib

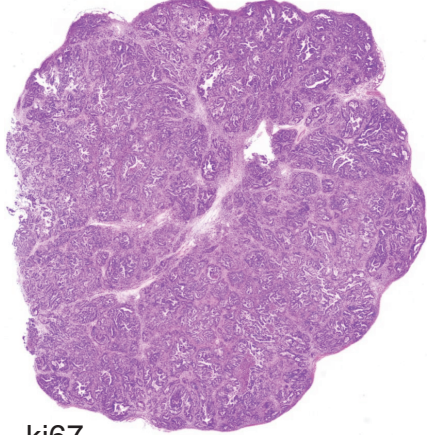**B****H&E**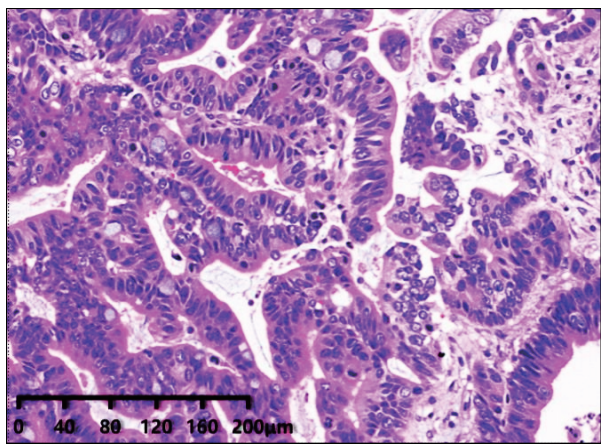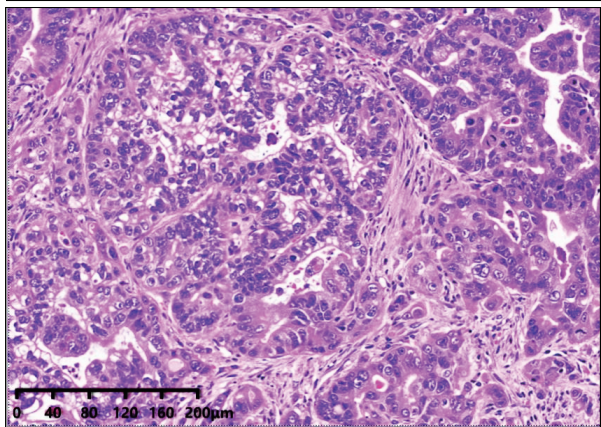**C****ki67****ARNTL2****EGFR**

Vehicle

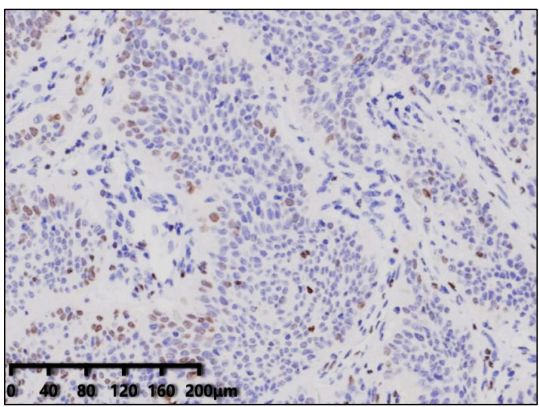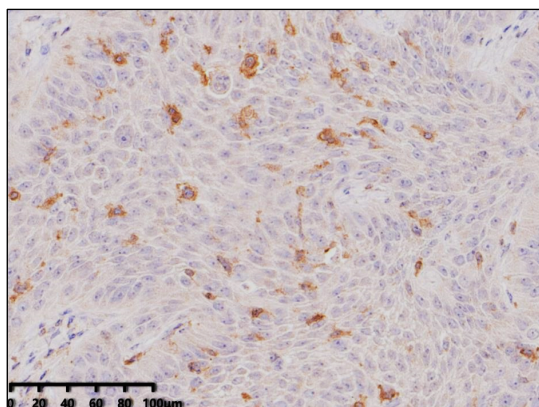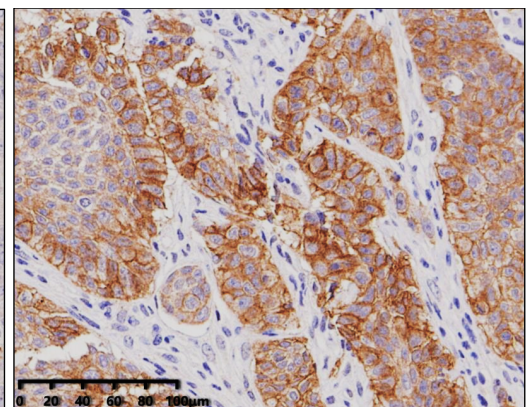

Erlotinib

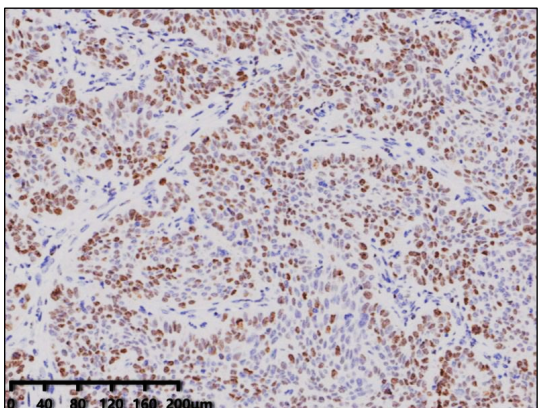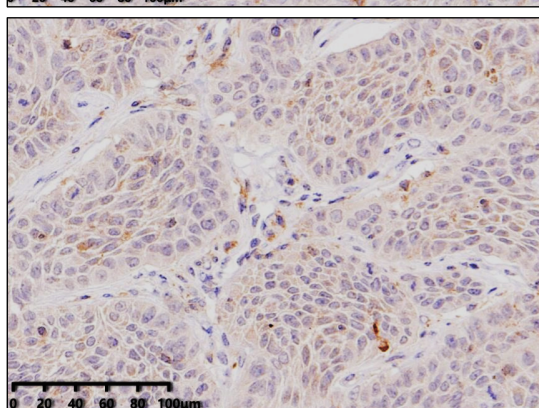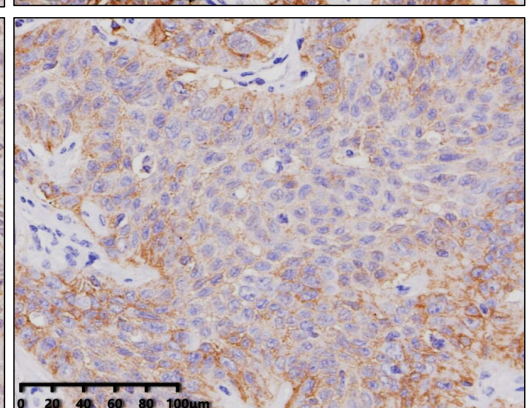

Supplement: Supplementary file 1 [file 12943_2024_1965_MOESM1_ESM.zip › S3.pdf]
